# Multifunctional Finishing of Cotton with Compounds Derived from MCT-β-CD and Quantification of Effects Using MLR Statistical Analysis

**DOI:** 10.3390/polym13030410

**Published:** 2021-01-27

**Authors:** Vasilica Popescu, Marioara Petrea, Andrei Popescu

**Affiliations:** 1 Department of Chemical Engineering in Textiles and Leather, “Gheorghe Asachi” Technical University of Iasi, 700050 Iasi, Romania; 2 Department of Wood Processing and Design of Wood Products, “Transilvania” University of Brasov, 500068 Brasov, Romania; gmaria@unitbv.ro; 3 Department of Machine Design, Mechatronics and Robotics, “Gheorghe Asachi” Technical University of Iasi, 700050 Iasi, Romania; andrei.popescu@academic.tuiasi.ro

**Keywords:** ethylenediamine, glyoxal, MCT-β-CD, multifunctional cotton, spectroscopy, wrinkle recovery angle, hydrophilicity, antibacterial capacity, quality indices, statistical analysis

## Abstract

Multifunctionalization of cotton using a single product has not been made until now. Such a product was synthesized using compounds with multiple functions (glyoxal, ethylenediamine (ED) and monochlorotriazinyl-β–cyclodextrin (MCT-β-CD)), under different mass ratios. Obtaining this multifunctional derivative has been confirmed by spectroscopic analyses (^1^H-NMR and FTIR) and a scanning electron microscope (SEM). Treatment of cotton with the MCT-β-CD derivative (D-CD) has been realized with the pad dry-cure technology. The presence of this multifunctional derivative on cotton was highlighted with spectroscopic (FTIR, EDAX, XRD) and thermoanalytical (DSC) methods. The objective of treating cotton with D-CD was to achieve four simultaneous effects: large wrinkle recovery angle (WRA), hydrophilicity, antibacterial capacity and a good breaking resistance. This objective has been achieved, so the garments that will be manufactured with such multifunctional cotton will be more comfortable. The efficiency of treatments with D-CD was marked out by multiple linear regression (MLR) and certain quality indices. Using MLR, the behavior of the treated cotton was mathematically modeled and the stationary/optimal points corresponding to each effect were calculated. Quality indices have been calculated and all final samples had values higher than 1, which confirmed the positive effects exerted by D-CDs on cotton.

## 1. Introduction

Multifunctional cotton fabric can be made by chemical treatments performed by the following techniques: (1) successive treatment (layer-by-layer); (2) classic treatment, in a single stage (single-layer) [1]. Using the layer-by-layer technique, the cellulosic materials were functionalized in two or more stages, leading to a wide range of effects, as follows:

1. Wrinkle-free, antibacterial, flame retardant and antioxidant properties on linen fabrics due to a finishing with chitosan-citric acid and phytic acid-thiourea [2];
2. Wrinkle-free, antibacterial, flame retardant, UV protection and antioxidant properties using layer-by-layer finishing with chitosan, sodium lignin sulphonate and boric acid [3];
3. Biocidal and hydrophobic properties when the cotton fabrics were modified with difunctional polysiloxanes [4];
4. Water repellence, flame retardance and antibacterial properties through deposition of three-dimensional tetrakis (hydroxymethyl) phosphonium chloride-urea polymer coating [5];
5. Crease resistance in addition with the antimicrobial effects on knitted fabric using dimethylol dihydroxy ethylene urea (DMDHEU) and titanium dioxide (TiO_2_) [6].

Multiple and diverse effects were obtained on cotton by finishes based on the single-layer technique, as follows:

1. Water repellent, stain repellent, shrink resistance and quick dry properties using fluorocarbon resin [7];
2. Wrinkle recovery, antibacterial effect, ultraviolet protection, bending rigidity and antistatic properties using butanetetracarboxylic acid and zinc oxide nanoparticles [8];
3. Biocompatibility with durable antibacterial properties using ZnO nanoparticles and gallic acid [9];
4. Flame retardance, antibacterial and water repellence effects were obtained via a chemical foam application method [10];
5. Coloration, antioxidant and antimicrobial effects on cotton fabrics using pomegranate peel extract and silver nanoparticles synthesized by a green biochemical reduction method [11];
6. Fireproof, anti-soil, oleophobization and anti-crease effects using a mixture of phosphorus compound, a perfluorinated resin and melamine compound [12];
7. Antibacterial activity, electrical conductivity, superhydrophobicity, catalytic activity, ultraviolet blocking and coloration properties using *N*-(2-ethylhexyl) carbamate aqueous solution, followed by microwave-assisted reduction of silver ions (Ag^+^) on the fibrous cotton surface [13].

All these studies indicate that the chemical component of the treatment agents and especially their reactivities determine the diversity and number of effects obtained during the multifunctional finishing of cotton. Conferring antibacterial effects simultaneously with various other effects was possible by using classic/ecofriendly finishing compounds in the presence/absence of some biocides (TiO_2_, silver ions or nanoparticles, zinc oxide nanoparticles) [2,3,4,5,6,8,9,10,11,13]. The combination of wrinkle-proofing and hydrophilicity effects on cotton is extremely difficult to achieve even when using compounds with increased reactivity and functionality (such as glyoxal and MCT-β-CD), commonly used in wrinkle-proofing treatments.

Glyoxal, ethylenediamine (ED) and MCT-β-CD are substances with high reactivity caused by the presence of at least two functional groups, identical or different (in the case of MCT-β-CD). Glyoxal was used in chemical modification of cellulose as a crosslinking agent [14] or as a non-formaldehyde wrinkle-proofing agent or durable press agent [15,16,17,18,19]. The obtained results were good, being close to those obtained when using the classical formaldehyde-type (urea—or melamine-formaldehyde) products. However, the cotton treated with glyoxal presented the shortcoming of a poor water absorbency capacity [15].

Even if glyoxal utilization in combination with certain agents carrying polar groups (glycol, diethylene amine, triethylene amine, vinyl alcohol, acryl amide, chitosan) represented the object of numerous studies, water absorbency of the treated cotton has not been tested [16,20,21,22,23,24,25,26,27,28,29,30,31,32,33]. According to Laga and Wasif (2013) the values of the wrinkle recovery angle (WRA), tensile strength, whiteness index and abrasion resistance depend on the treatment conditions: temperature, duration of condensation, catalyst nature, concentration and the type of crosslinking agents and additives.

The product obtained from the chemical reaction of two difunctional compounds, glyoxal and ED, has been the subject of many scientific papers [24,25,26,27,28,29,30]. This product is piperazino-piperazine type or Schiff base, depending on the working conditions. However, the piperazino-piperazine compounds were not applied on cotton, although they were water-soluble. The imines have not generated simultaneous effects as wettability and wrinkle-proofing when they were applied on cotton.

Imines were also obtained from the reaction of a diamine having a long alkyl chain, with a dialdehyde obtained from the oxidation of cellulose. In this case, the crosslinking phenomenon was accompanied by a strong hydrophobic effect and a low hygroscopicity for the cellulosic material [31].

Contrary to expectations, application of some multifunctional products (MCT-β-CD via BTCA) on cotton did not improve water absorbency; yet, it resulted in good wrinkle-proofing effects. The explanation was based on the appearance of a strong network between those multifunctional products and cellulose [32]. It is known that the utilization of a polycarboxylic acid (BTCA) for cotton finishing is not the optimum solution, because high-temperature treatment can affect both the whiteness index and the integrity of the textile support.

However, obtaining four simultaneous effects refined the treated textile support was reported only in one of our previous work [33]. We have obtained very good values for WRA, hydrophilicity, durability at repeated washing as well as tensile strength by treating the cotton with three agents; we applied the pad-dry-cure technology three times: the first agent was a polyol, then a non-formaldehyde wrinkle-proofing agent (chitosan) and finally, a multifunctional product with many polar groups (MCT-β-CD).

The novelty brought by this article consists in the generation of multiple effects on cotton (as wrinkle-proofing, hydrophilicity, antibacterial capacity and preserving the textile support integrity unaltered) using a synthesized compound, rich in different functional groups (OH, NH_2_). In this article we hypothesized that the realization of these multiple effects is possible on a cotton fabric using a single compound, even if these effects are usually contradictory. This work proves that these effects become simultaneous only when adequate multifunctional compounds are used, such as MCT-β-CD derivatives (D-CD). These derivatives were obtained in two steps: (1) obtaining a Schiff base from the interaction between glyoxal and ED; (2) the reaction of the Schiff base with MCT-β-CD. The high functionality of D-CD is due to the presence of many NH_2_, C=O and OH groups. The D-CD was studied by spectroscopic analyses (^1^H-NMR, FTIR). The presence of new functional groups on cotton, after treatment, was highlighted with FTIR, SEM, EDAX, XRD and DSC analyses. The presence of these four simultaneous effects leads to an increase of the comfort index of the cotton fabric; the clothes realized with this multifunctional cotton fabric can be easily cared for and can be worn immediately after washing and drying, without a prior smoothing; in addition, they are resistant to tearing and attacks from certain bacteria. The treatments with D-CD ennoble the cotton and clothing products manufactured from it.

## 2. Materials and Methods

The MCT-β-CD product (monochlorotriazinyl–β-cyclodextrin or 6-(2-chlor-4-hydroxy)-1,3,5-triazenyl-beta-cyclodextrin, under trading name Cavasol W7 MCT) was obtained from Wacker-Chemie Company, Munchen, Germany, its chemical structure is presented in the supplementary material (Table S1). CAVASOL^®^ W7 MCT is a reactive β-cyclodextrin derivative with an average molecular weight of 1560 g/mole and a substitution degree (i.e., units’ number of triazine substituent per glucopyranosyl unit) equal to 0.4; the substitution degree per mole of β-cyclodextrin is equal to 2.8 [34,35,36,37,38]. A detailed characterization of this product is made in the supplementary material (Figure S1 and Table S2). The intermediate agent/Schiff base and D-CD have been characterized by spectroscopic methods (^1^H-NMR and FTIR). The substances glyoxal, ED, NaOH and Na_2_CO_3_ were acquired from Merck Company, while the non-ionic surfactant (Romopal O) was acquired from Bega Chim SA Company from Timisoara, Romania. Glyoxal is marketed as 40% solution (i.e., wt% in H_2_O).

The 100% cotton fabric was obtained from a Romanian company and had 145 g/m^2^ weight. The cotton fabric was prepared by scouring with 2% owf (percent on weight of fabric) NaOH, 1% owf Na_2_CO_3_, 1% owf Romopal O, liquor ratio M = 50:1, at the temperature of 100 °C for two hours. This was followed by a hot and then a cold rinsing and drying at room temperature.

Firstly, we synthesized the D-CDs using two steps: (1) interactions of 3–15% owf glyoxal with 3–15% owf ED for 30 min, under strong stirring led to Schiff bases; (2) Interactions of Schiff bases with 6–9% owf MCT-β-CD led to the obtaining of D-CDs. This step was also performed 30 min under vigorous stirring using a magnetic stirrer. Finally, the D-CDs were applied on the cotton samples. From the fabric prepared by scouring, one was realized by three series of 20 samples, taken both on warp and weft directions. Each cotton sample treating with a D-CD was performed by applying the pad-dry-cure technology, as follows: (1) Padding with the D-CD solution in the presence of a catalyst (NaOH, pH = 12) was realized on a mini padder, using 100% squeezing degree. (2) Drying realized at 100 °C for 4 min; (3) Polycondensation was made at 150 °C for 4 min. For drying and polycondensation, we used a minitherm device type ERNST BENZ AG. Final samples were washed and dried at room temperature.

### 2.1. Statistical Analysis

To perform the statistical analysis, we used multiple linear regression (MLR) software with three factors/independent variables. MLR can determine the mathematical equations that indicate the correlation between the dependent variables Y (i.e., any effect obtained through treating cotton) and the independent variables (X_1_, X_2_ and X_3_, i.e., the percent of glyoxal, ED and MCT-β-CD, respectively, on weight of fabric). MLR can find the coefficients (b_i_ = b_0_, b_1_...b_33_) of some predefined Equation (1) [39,40,41]:

(1) $$Y=b_{0}+b_{1}X_{1}+b_{2}X_{2}+b_{3}X_{3}+b_{12}X_{1}X_{2}+b_{13}X_{1}X_{3\;}+b_{23}X_{2}X_{3}+b_{11}X_{1}^{2}+b_{22}{\;X}_{2}^{2}+b_{33}X_{3}^{2}$$

MLR offers both qualitative information and, especially, quantitative information: it indicates the presence of stationary points (maximum point when coefficients b_11_ < 0, b_22_ < 0, b_33_ < 0 or minimum point when b_11_ > 0, b_22_ > 0 and b_33_ > 0) and quantifies their values by annulment of the first order derivatives of each mathematical model [39,40,41].

The coefficients’ signs of X_1_, X_2_ or X_3_ indicate the influence direct (when b_1_ > 0, b_2_ > 0, b_3_ > 0) or inversely proportional (only for the factor for which the coefficient b_i_ < 0, i = 1 ÷ 3).

Each mathematical model obtained by us using MLR was validated with two tests: Student’s *t*-test (to verify the significance of each regression coefficient) and Fisher’s test (to verify the adequacy of each mathematical model) [42,43]. MLR was used to find the combinations between X_1_, X_2_ and X_3_ that led to maximal wrinkle-proofing effects (WRA_dry_ and WRA_wet_) and water absorption capacity.

In this work, the treating agent (D-CD) was obtained as the result of the chemical interaction between the chemical compounds that have at least two reactive groups: a di-aldehyde (glyoxal), a diamine (ED) and a multifunctional cyclic oligosaccharide (MCT-β-CD). For D-CD synthesis, we used 5 working amounts (Table 1 and Table 2) for these substances.

In Table 1 and Table 2, the significances of the three independent variables are as follows: X_1_ represents the percent on weight of fabric (% owf) in case of 40% glyoxal solution; X_2_ represents the ED percent on weight of fabric; X_3_ is MCT-β-CD percent on weight of fabric.

In order to find the optimum amounts of each reacting partners that give a D-CD able to produce multiple effects on cotton, we resorted to planning the manner to combine the three substances, according to a working plan required by multiple linear regression (MLR) [39,40], with three independent variables (Table 1 and Table 2). The experimental design was performed using a complex centered rotatable program that had 6 experiments in the center of the experimental region and 20 total experiments. Table 2 presents both reaction conditions in order to obtain the D-CD and the utilized codes: (1) individual coding of each D-CD (i.e., D1 to D20); (2) coding of final cotton samples (S1–S20).

### 2.2. Proton Nuclear Magnetic Resonance (^1^H-NMR)

The ^1^H-NMR spectra for individual reagents and their mixture (after 30 min magnetic stirring) were recorded on a Bruker Avance DRX 400 MHz Spectrometer (Bruker BioSpin GmbH, Rheinstetten, Germany) equipped with a 5 mm QNP direct detection probe and z-gradients. Spectra were recorded in D_2_O at room temperature. The spectra were referenced relative to the solvent residual peak (4.8 ppm).

### 2.3. Fourier Transform Infrared Spectroscopy (FTIR)

FTIR analyses were performed both for the synthesized agent (D14) and the treated cotton sample. A Multiple Internal Reflectance Accessory (SPECAC, Fort Washington, PA, USA) with ATR KRS-5 crystal of thallium bromide-iodide, having 25 reflections and the investigation angle of 45 degrees, was used. This accessory device was attached to the Spectrophotometer FTIR IRAffinity-1 Shimadzu (Kyoto, Japan), the spectra registration being realized with 250 scans in the 4000–450 cm^−1^ range.

### 2.4. Scanning Electron Microscopy (SEM) and Energy Dispersive X-ray Spectroscopy (EDAX)

SEM and EDAX analyses of cotton samples were performed with an electron microscope type QUANTA 200 3D DUAL BEAM (FEI Company, Hillsboro, OR, USA). This microscope is a combination of two systems (SEM and FIB), by whose means, by sending an electron beam on the treated samples, three-dimensional images could be obtained, with a magnification of 100,000. Moreover, by using the X radiation with dispersive energy (EDAX), the elemental analyses were possible for the identification of the surface characteristics and a high-resolution chemical analysis.

### 2.5. X-ray Diffraction Analysis (XRD)

XRD analysis used a X’PERT PRO MRD X-ray generator (produced by PANalytical, Almelo, The Netherlands) with a copper target, for collection of intensity data. The identical experimental conditions were maintained for all the samples tested in this paper. Monochromatic Cu-Kα radiation (λ = 1.54 Å) was obtained using a 10 μm-thick nickel filter to irradiate cotton fibers packed in Mark capillary tube of 1 mm diameter.

The results were interpreted by X’PERT PRO MRD software (Almelo, The Netherlands) and in the end, the material diffractogram was obtained. The “Gaussian + linear” method was used to determine the crystallinity of five samples: control sample, S10, S12, S14 and S15.

This method is based on the Gaussian peak fitting with a linear background and includes a larger scattering angle region, from 2θ_1_ = 13° to 2θ_2_ = 50°. The crystallinity index (CrI) was calculated as the ratio between the area of the crystalline contribution and the area under the sample intensity curve [44]. CrI is dimensionless, but if the reporting is done at 100, then this index can be expressed as a percentage [45].

### 2.6. Wrinkle Recovery Angles (WRAs)

By using the apparatus Metrimpex FF-07 (Metrimpex Hungarian Trading Company, Budapest, Hungary) and complying with standardized German method DIN 53890, we determined the WRAs along warp/weft to compute the WRA on dry samples (WRA_dry_) and on wet samples (WRA_wet_) respectively.

### 2.7. Water Absorption Capacity

To perform the longitudinal wicking test, we used three series of treated samples cut (along warp direction) at the size 25 × 200 mm. These were attached on the upper bar of the apparatus destined to the determination of absorption by capillarity. The initial point of the calibrated scale was brought at the level of the solution. In this way, at the bottom side of textile stripe there were 3 cm of each sample immersed into a solution of 0.2% eosin (yellow acid dye) at the temperature of 20 °C. We measured the rising height of the solution every 5 min, until the moment when the level remained constant. Each result is the mean of three determinations.

### 2.8. Differential Scanning Calorimetry (DSC)

The DSC curves have been recorded with a Mettler Toledo DSC1 apparatus in inert atmosphere (nitrogen) at a flow rate of 150 mL/min, with a heating rate of 10 °C/min. The mass of the analyzed sample was around 2.5 mg. Crucibles were made of aluminum and had capacity of 40 μL (Al crucibles 40 μL with pin). An empty pan was used as reference. The operational parameters were maintained constant for all the tested samples, in order to obtain comparable data.

### 2.9. Integrity of Textile Support

The textile support integrity was assessed according to the value of breaking strength of the treated sample. According to EN ISO 13934-1/1999 standard, the breaking strength of textile support was determined both along the warp and weft direction. A dynamometer H5K-T Tinius Olsen type for yarns and textiles was used, endowed with computer software QMAT TEXTILE (Tinius Olsen Testing Machine Company, Horsham, PA, USA).

### 2.10. Quality Index, I_C_

Any total crosslinking process causes a reduction in the integrity of the textile support, which is observable by obtaining lower values for breaking strength of the samples treated comparative with the standard. By using the breaking strength values, the quality index (I_C_) was calculated; I_C_ correlates with the force necessary for breaking the fabric, with WRA, according to Equation (2) [46,47]:

(2) $$I_{C\;}=\frac{3\;\times {\;F}_{\mathrm{treated}\;\mathrm{sample}}\;\times {\;\mathrm{WRA}}_{\mathrm{dry}}}{F_{\mathrm{control}\;\mathrm{sample}\;}\times \;\left(180+{\;\mathrm{WRA}}_{\mathrm{control}\;\mathrm{sample}\;}\right)}$$

where: F_treated sample_ = breaking strength on a direction (warp or weft) of the treated sample, in N;

F_control sample_ = breaking strength on a direction (warp or weft) of the control sample, in N;

WRA_dry_ = wrinkle recovering angle on a direction (warp or weft) of the dry treated sample, in degrees; WRA_control sample_ = wrinkle recovering angle on a direction (warp and weft) of the untreated sample, in degrees.

The values of Ic are assessed as follows:

I_C_ = 1, wrinkle-proofing treatment is considered good, because the realized value of the WRA, and the loss of breaking strength ranges between admitted limits;

I_C_ > 1, the treatment is considered very good, the loss of breaking strength being very small in regard to the value of the obtained WRA;

I_C_ < 1, wrinkle-proofing treatment is considered unsatisfactory, because the increase of the value of WRA results in the diminution of breaking strength beyond the admitted limits.

### 2.11. Antibacterial Capacity

The diffusimetric method has been used for antibacterial capacity testing. Two types of microorganisms from the collection of Biology Laboratory of DTNW Krefeld, Germany have been used as follows: A Gram-positive coccus (*Micrococcus luteus* ATCC 934) and a Gram-negative bacillus (*Escherichia coli* DSMZ 498). The diffusimetric method consisted of the application of textile disks (15 mm) on the surface of a bacterial culture realized in agar (same procedure as in our previous work) [48]. Antibacterial capacity was tested on two series of samples: (1) samples S4 and S8; (2) samples S12 and S11; the two series were tested both in absence and in presence of 5 g/L AgNO_3_ solution (at 20 °C for 5 min). All samples were washed (repeatedly for 0, 5 or 10 times) according to ISO 105-C10:2006, test C (60 °C, 30 min).

## 3. Results and Discussion

### 3.1. Synthesis and Characterization of D-CD

The selection of three chemical substances (40% glyoxal solution, ED and MCT-β-CD) for the synthesis of D-CD was based on several considerations:

1. Glyoxal was chosen to confer good wrinkle recovery capacity to the cotton, because glyoxal has high reactivity and high polymerization capacity [17,49,50]. Glyoxal has a high capacity to react with water, giving reactions of hydration, oligomerization and disproportionation [26,27,51,52,53,54,55]. Glyoxal solution is a mixture between the non-hydrated form (glyoxal in the form of CHO-CHO, of trans-type) and the hydrated forms of monohydrate and dihydrate types (gem-diol, dimer and trimer) (Table S1).
2. ED was chosen because it reacts easily with glyoxal, giving condensation reactions [56,57];
3. MCT-β-CD is a multifunctional compound that could form ether links with cellulose from cotton fabric [58,59,60]. Due to the big number of OH groups disposed to the outside of the truncated cone form, and to the large volume occupied by MCT-β-CD and D-CD respectively, the treated cotton is expected to have good absorption of aqueous solutions.

**Scheme 1:**
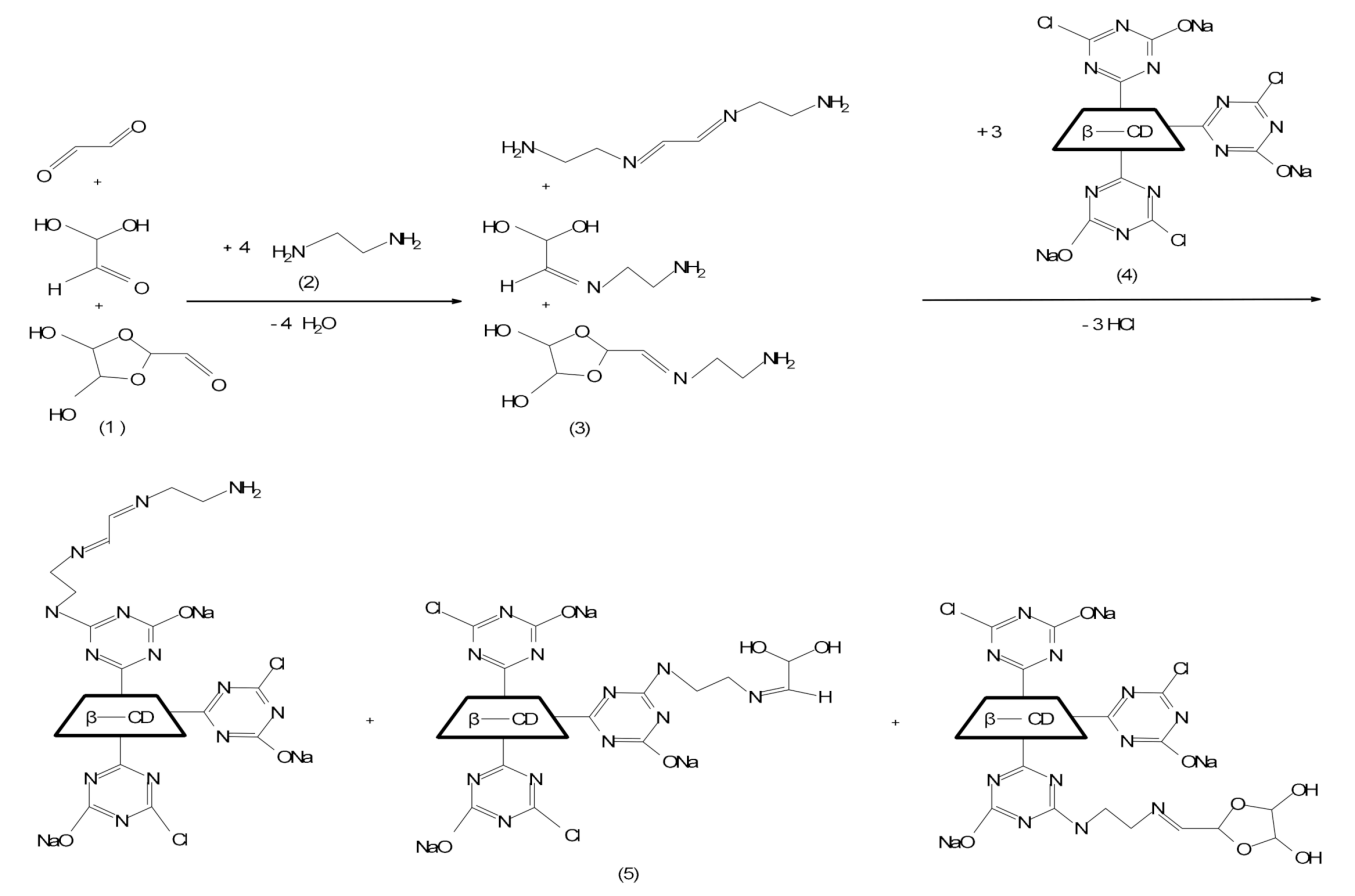
Monochlorotriazinyl-β–cyclodextrin derivative (D-CD) synthesis: (**1**) species (monomer (M) and monohydrated (MH)) in glyoxal solution, (**2**) ethylendiamine (ED), (**3**) Schiff bases, (**4**) monochlorotriazinyl-β–cyclodextrin (MCT-β-CD), (**5**) D-CDs.

The synthesis of each D-CD consisted of combining the reagents in the following manner:

- Mixing the volume of glyoxal with that of ED in a Berzelius beaker (according to data in Table 1 and Table 2) and stirring for 30 min. This step leads to the generation of imines/Schiff base; it is known that a glyoxal solution is a mixture of monomer (M), monohydrated (MH) and dehydrated (DH) forms. In this step, only M and MH can react with ED, as in Scheme 1.
- Adding NaOH (as catalyst, up to pH = 12) in the same Berzelius beaker where the Schiff base was obtained; after vigorous stirring (30 min), the MCT-β-CD solution was added (according to the data in Table 1 and Table 2) and the stirring was continued for 30 min at room temperature to allow the *N*-alkylation reactions to take place, with the formation of the D-CDs compounds.

Literature indicates that the reaction between glyoxal and ED depends on the nature of amine, temperature, presence/absence of a catalyst and on the molar ratio of glyoxal:amine [24,25,26,27,28,29,30]. The utilization of diamine in excess (ED) in a molar ratio glyoxal: ED of 1:2 or 2:3 (at T ≥ 90 °C, in the presence of a buffer (NaH_2_PO_4_) for pH = 9), in aqueous or alcoholic solution, results in the generation of piperazino-piperazines (i.e., naphthopiperazine, 2, 3-diamino ethylene piperazine/decahydropiranozo 2, 3 b pyrazine) [24,25]. This product precipitates, being a white, crystalline solid, water soluble and with a slightly basic character; however, the piperazino-piperazine product was not applied on cotton.

At room temperature, as in this paper, the combination of glyoxal with ED occurs according to a nucleophilic addition reaction, followed by elimination, generating a Schiff base (or *N*-substituted imine) [26,27,28,29,30,61].

These imines have not generated simultaneous wettability and anti-creasing effects when applied on cotton. For this reason, we have used MCT-β-CD, a multifunctional compound able to react with Schiff base (generating D-CD), but keep some free functional groups to be able to react with cellulose.

Two spectroscopic analyses (^1^H-NMR and FTIR) were used to perform the characterization of the D-CDs.

**Figure 1:**
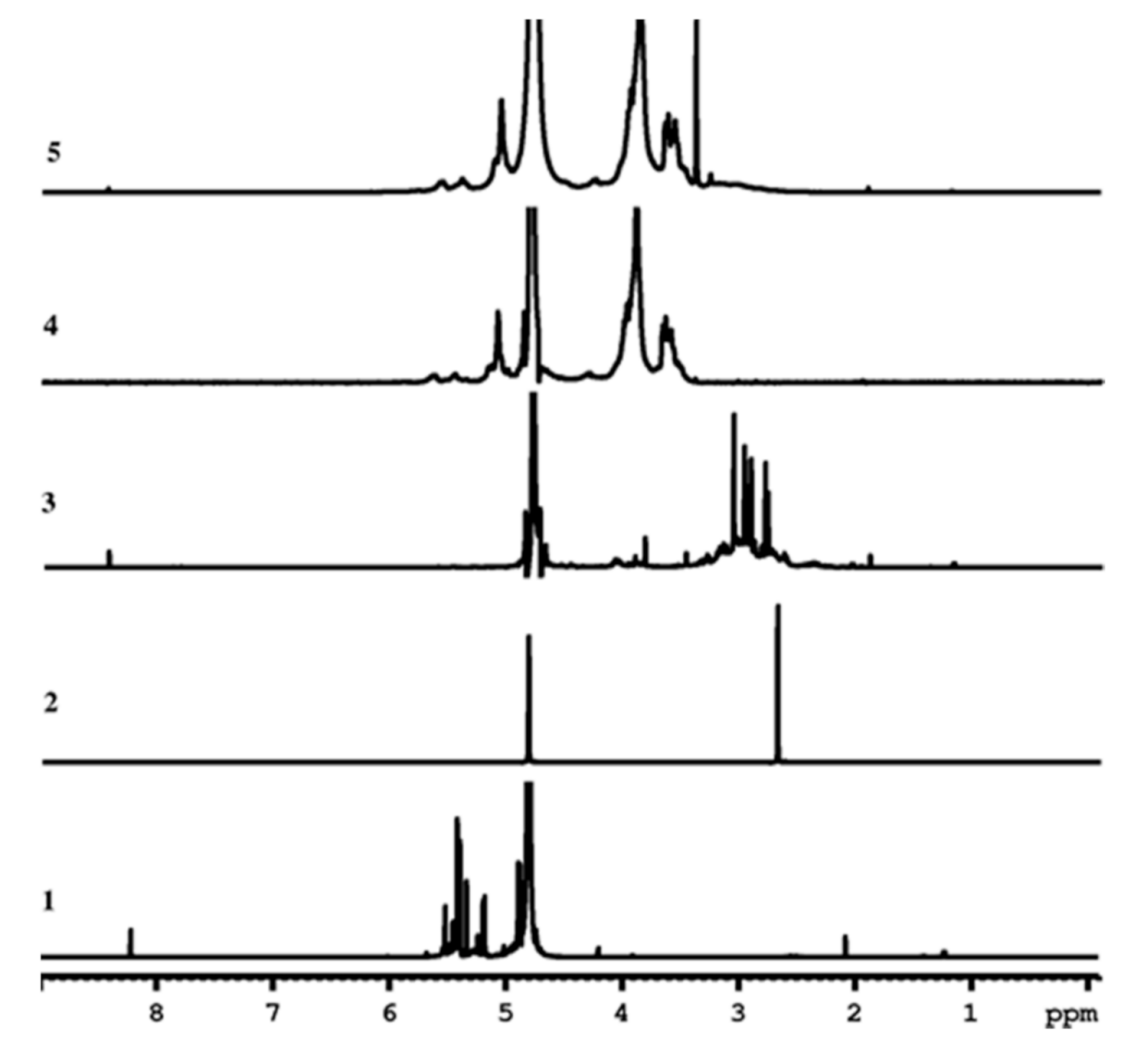
^1^H-NMR spectra (D_2_O, 400 MHz) between 9 and 0 ppm chemical shift, for glyoxal (**1**); ED (**2**); Schiff base, the intermediate compound obtained during interactions of glyoxal and ED in 1:2 molar ratio after 30 min of reaction (**3**); MCT-β–CD (**4**); D-CD (obtained during interactions of Schiff base and MCT-β-CD) (**5**).

The ^1^H-NMR spectra were carried out both for D14 (a D-CD), and for each reagent (or combination of two or three reagents) used in the synthesis of D14 (Figure 1).

The ^1^H-NMR spectrum of the glyoxal solution (Figure 1) indicated both the presence of the non-hydrated (CHO–CHO) and monohydrated (CHO–CH(OH)_2_) monomer, respectively, at 8.22 ppm chemical shift, and of some oligomers (dihydrated forms of dimer and trimer type) at 4.87–6.0 ppm [62].

The ^1^H-NMR spectrum of ED contained two peaks: 4.8 ppm for the solvent (D_2_O) and 2.68 ppm for CH_2_.

The ^1^H-NMR spectrum recorded 30 min after the combination of glyoxal solution with ED (at a molar ratio glyoxal:ED = 1:2) showed that the reaction between the two reagents really occurred, and the peaks corresponding to glyoxal and ED have disappeared, because a new product was formed (Figure 1). In fact, it was a mixture consisting of the imines obtained from ED reaction with aldehyde groups from the monomer, and, respectively, from the oligomer that contained a CH=O group (namely from (1, 3) dioxalane-4,5-trans-diol, Table S1). The presence of Schiff base is confirmed by δCH from 8.44 ppm [63,64].

For MCT-β-CD, the ^1^H-NMR spectrum (Figure 1) contained chemical shifts for the solvent (D_2_O, at 4.796 ppm) and broad peaks for the protons from the glucopyranosyl units from cyclodextrin at 3.4–3.8 ppm (H2 and 4), 3.8–4.4 ppm (for H3, 5 and 6) and 4.9–5.6 ppm (anomeric protons).

The ^1^H-NMR spectrum obtained for D-CD shows that the peaks corresponding to the imine formed in the first step (when glyoxal was combined with ED) were present in the final product (at 8.433 ppm). The presence of CH_2_ group from ED was confirmed in the D-CD by δCH from 3.38–3.015 ppm. The presence of cyclodextrin in this D-CD was very clear, because this spectrum had a form very similar to that of the MCT-β-CD solution (Figure 1). The ^1^H-NMR results confirmed the chemical structures shown in Scheme 1.

**Figure 2:**
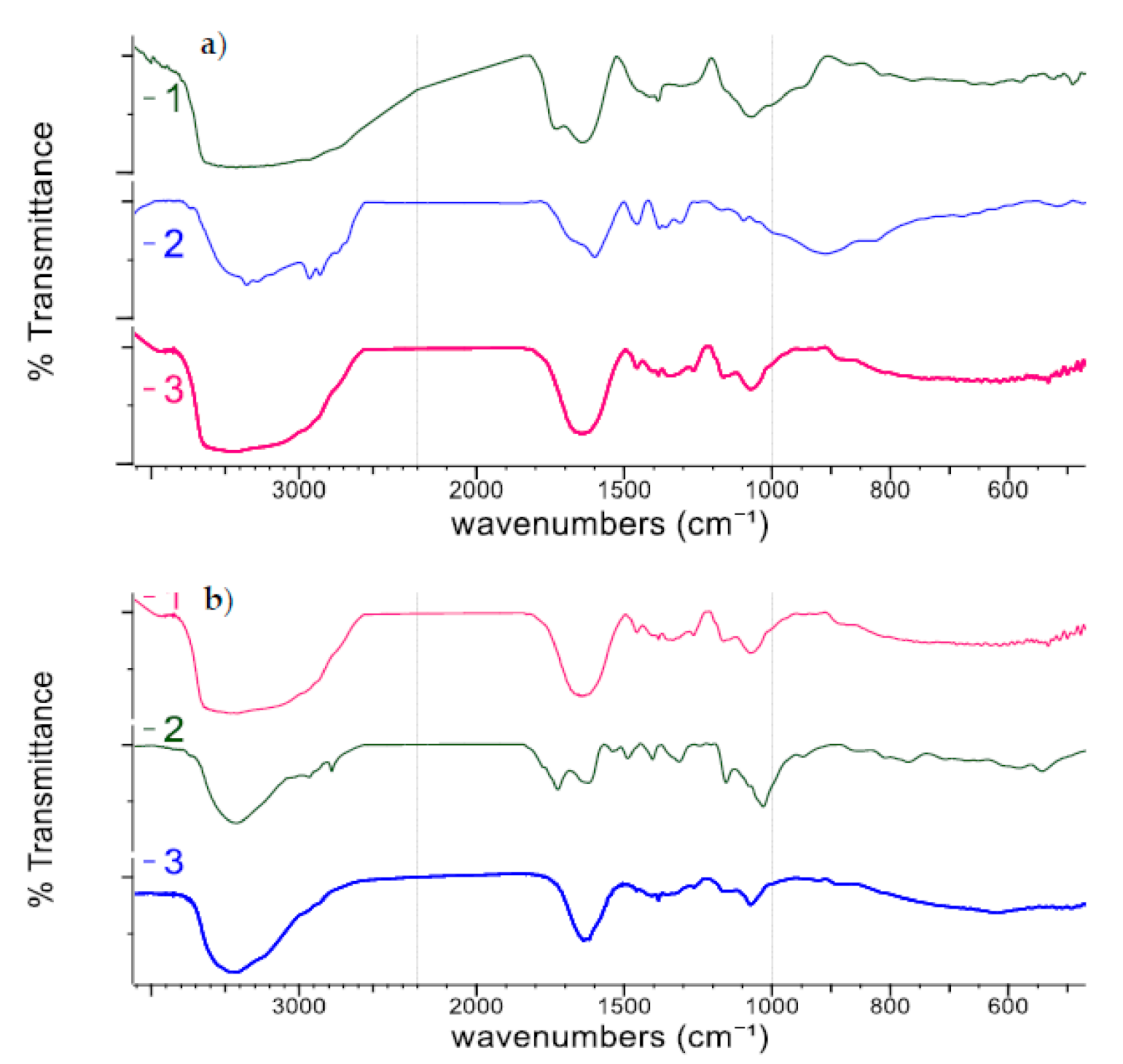
FTIR spectra for: (**a**) compounds involved in obtaining the Schiff base: glyoxal solution (**1**), ED (**2**) and the Schiff base/intermediate agent obtained from glyoxal interaction with ED (**3**); (**b**) compounds involved in obtaining the D-CD: Schiff base (**1**); MCT-β-CD (**2**) and D-CD (D14) (**3**).

The FTIR spectrum of D-CD (D14, as in Table 2) indicated the presence of OH, NH_2_, C=N and C–N groups (Figure 2) as the result of the interaction between the three multifunctional substances: 40% glyoxal solution, ED and MCT-β-CD.

Figure 2 shows the following aspects:

- The presence of the Schiff base was confirmed by the disappearance of the absorption band specific to C=O from glyoxal of monomer type (at 1727 cm^−1^), as the result of transformation of this group in C=N. The absorption band for C=N appears at 1644 cm^−1^ (Figure 2a) [65]. However, ED, being a bifunctional compound, has two NH_2_ groups, of which only some are converted to C=N groups, and others remain non-involved in this chemical reaction. This fact was confirmed by the peaks from 3416–3413 cm^−1^ (NH_2_ asymmetric and symmetric), 1644 cm^−1^ (N–ppH bend overlapped on C=N) and 1073 cm^−1^ (C-N stretch).
- The presence of some absorption bands specific to primary amines on the spectrum of D-CD (D14, Figure 2b) was confirmed by NH_2_ in-plane bend from 1637 cm^−1^ and two NH_2_ stretchings (asymmetric and symmetric) at 3478 and 3417 cm^−1^ (overlapped on OH stretching derived from glyoxal and MCT-β-CD). This fact proved the presence of NH_2_ groups non-involved in chemical interaction between the intermediate compound (Schiff base) and MCT-β-CD. These groups conferred good antibacterial capacity to cotton treated with D14 or with any D-CD synthetized in this work.
- The presence of MCT-β-CD in the final treatment agent (Figure 2b) was hard to notice, because the weak absorption band from 1530 cm^−1^, specific for C=N from triazine cycle, was covered by the large absorption band C=N from Schiff base (at 1644 cm^−1^) [32,66,67,68]; this fact is evident only when the spectra were overlapped.
- The presence of oligomers (derived from 40% glyoxal solution) in D-CD was confirmed by the existence of peaks specific for dioxalane cycles from dimer (770–760 cm^−1^) [69,70,71] (Figure 2b).

### 3.2. Interaction of D-CD with Cellulose

**Figure 3:**
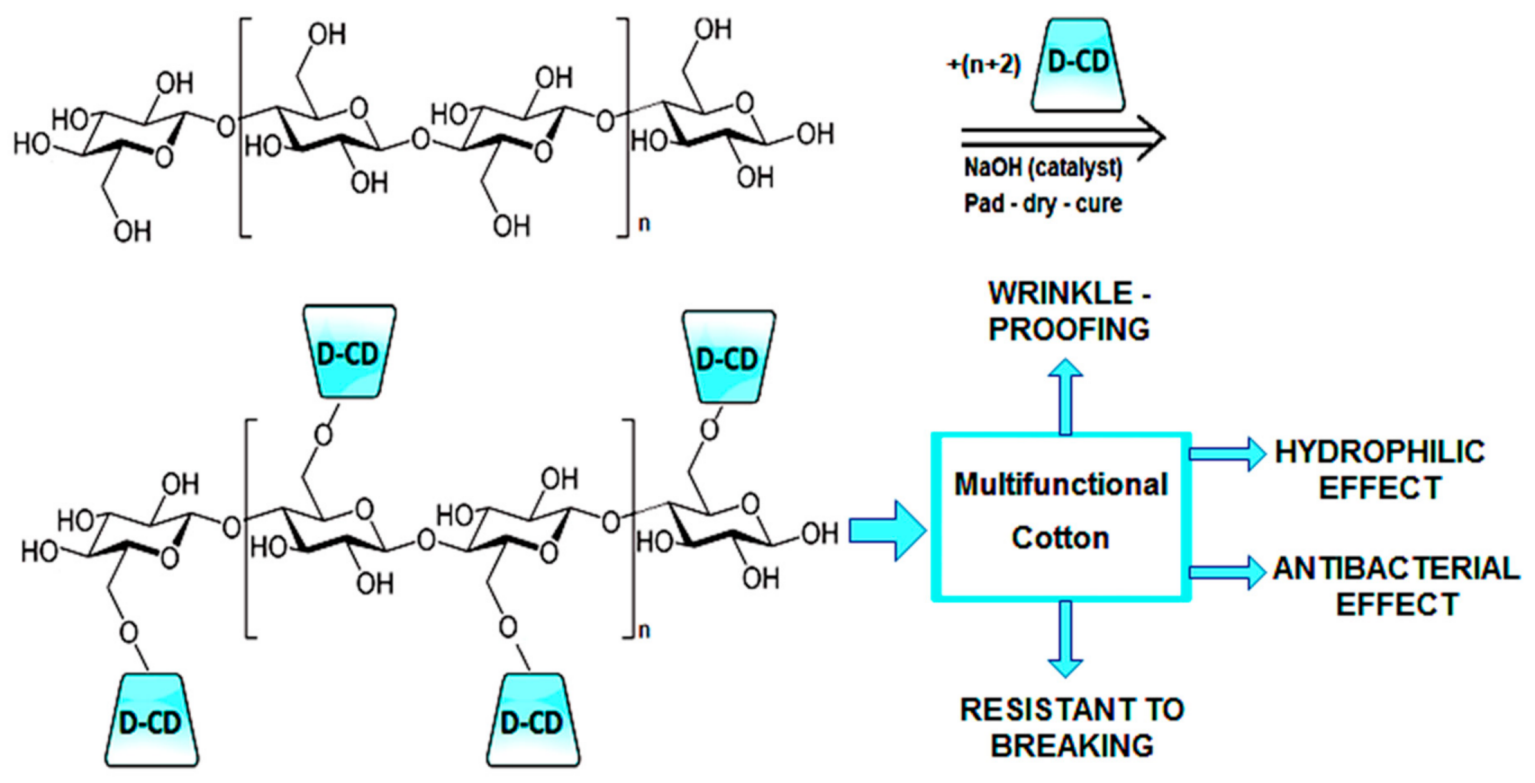
Getting multifunctional cotton (the chemical structures of D-CDs are shown in Scheme 1).

The realization of simultaneous effects was possible through the utilization of D-CD, according to Figure 3 and Figure S2. The presence of the catalyst was necessary for the realization of the reaction between cellulose and D-CD; the most suitable catalyst was NaOH, but it was not added again in the solution used for the cotton impregnation step, because it already existed there, being used as a catalyst since the processes of synthesis of D-CD compounds. NaOH determined the formation of ether bonds between the primary OH groups from cellulose and the chlorine atoms non-involved in reaction with Schiff base, but were attached on triazine rings from CAVASOL W7 MCT.

The mechanism was more complex because D-CD could be attached on the surface of the cotton sample (confirmed by SEM, EDAX and FTIR) and could penetrate inside cotton yarn (confirmed by XRD and by capillarity test). In fact, partial networks (partial crosslinkings) appeared both on the surface and inside cotton. The reaction between each D-CD (i.e., D1 to D20) and cellulose was possible due to the presence of NaOH as catalyst and the high temperature during the polycondensation stage from the adopted technology (pad-dry-cure).

In the case when D-CD has at least two free reactive groups, it can form ether bridges both with macromolecular chains of cellulose located in the cotton yarns and with another neighboring molecule of D-CD; D-CD becomes the bridge that strengthens the neighboring cotton yarns. This phenomenon is not uniform at all, and its appearance depends largely on working conditions during treating agent synthesis and pad-dry-cure technology process. Yet, it has a big influence on the wrinkle-proofing effect of cotton; this effect depends of type and number of bonds realized: chemical (covalent ether bridges) and physical (H bonds between the OH groups from β-cyclodextrin and cellulose). Mechanical deposits of D-CD in accessible areas of cellulose fiber can also influence the WRA values.

### 3.3. Characterization of Multifunctional Cotton

#### 3.3.1. Fourier Transform Infrared Spectroscopy (FTIR)

**Figure 4:**
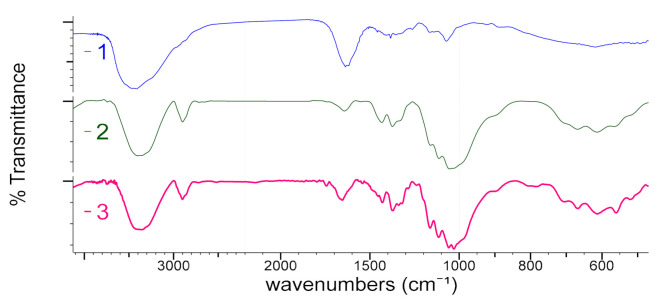
FTIR spectra for D-CD compound (**1**), control sample (**2**), treated sample (S15 as spectrum **3**).

FTIR spectra for the treated sample (S15) and for the standard sample are presented in Figure 4.

The presence of β-cyclodextrin in the S15 sample determines the increase of the peaks corresponding to OH (3351 cm^−1^), C–C (2895 cm^−1^) and C–O–C vibrations (1235 cm^−1^) [32,66,67]. Other significant peaks appeared due to the vibrations produced by C=N (between 1643 cm^−1^ and 1599 cm^−1^), C–N (1369, 1159, 1107 and 1027 cm^−1^) and NH_2_ groups (886 and 668 cm^−1^) (from D-CD). All this led to the appearance of higher peaks on the spectrum of treated sample comparative to the spectrum of untreated cotton. The etheric bond between cellulose and D-CD was confirmed by the increase of peak at 1235 cm^−1^. Numerous researchers confirm the belonging of the peak between 1643 and 1599 cm^−1^ to the Schiff bases (obtained from the monomeric glyoxal) [64,72,73,74]. The presence of MCT-β-CD (in D-CD) is confirmed by the absorption band from 1530 cm^−1^, specific for C=N from triazine cycle [74,75,76]. In sample S15, many OH groups corresponding to D-CD were transformed in COO^−^ groups, under alkaline medium realized with NaOH [52,53,77]. This fact is highlighted on spectrum 3, by the peak of 1729 cm^−1^.

#### 3.3.2. Scanning Electron Microscopy (SEM)

**Figure 5:**
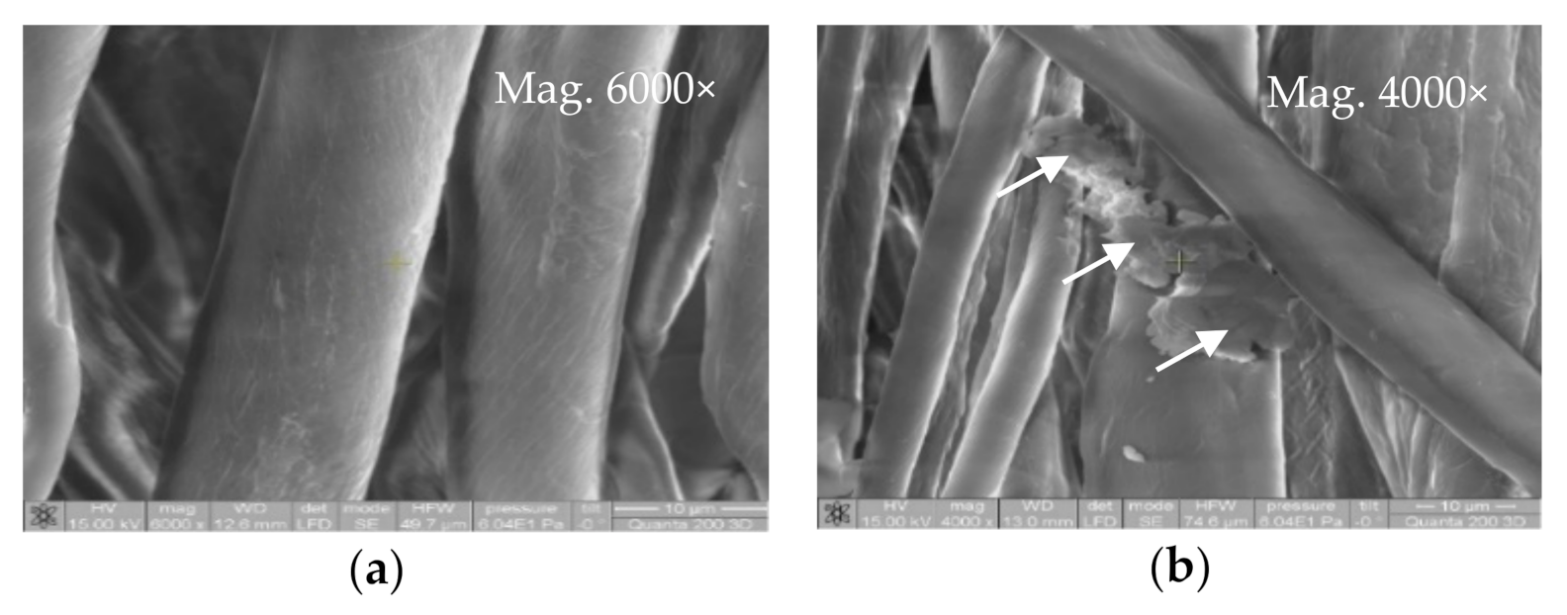

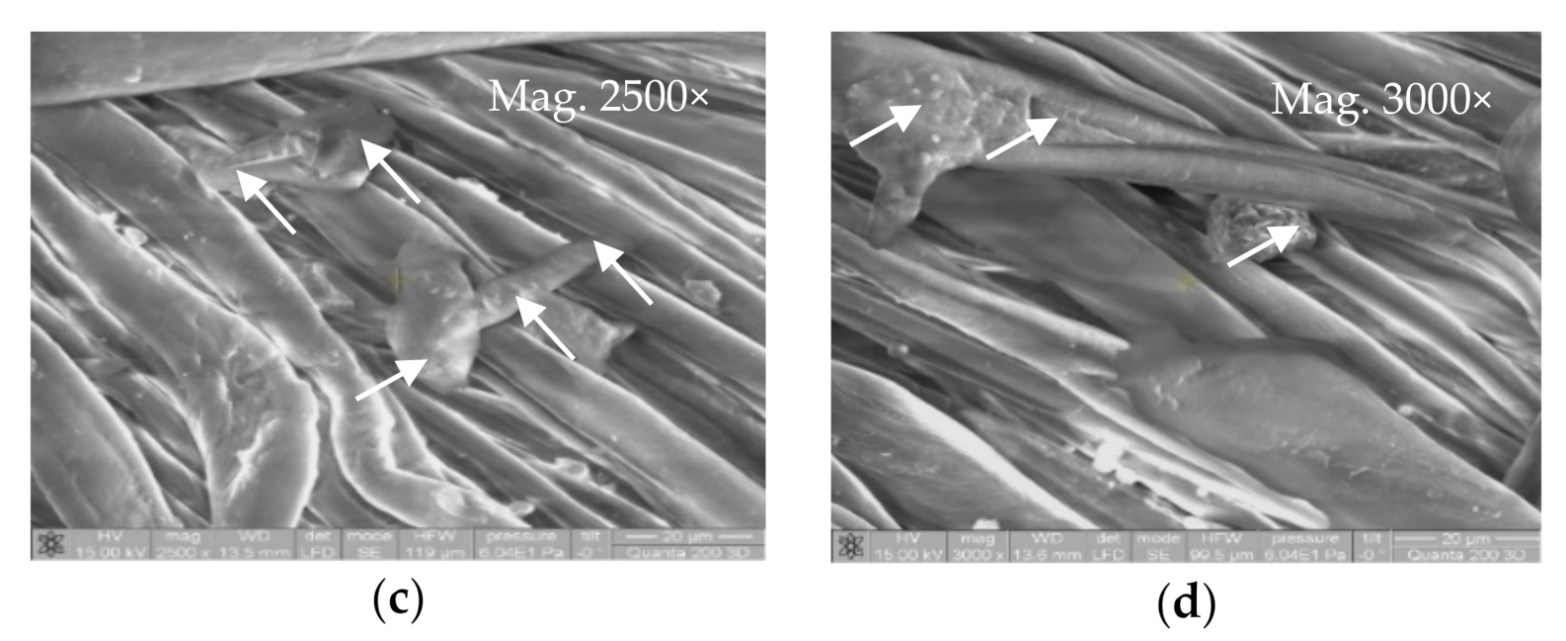
Morphologic aspect of some samples: (**a**) control sample; (**b**) S5 treated sample; (**c**) S14 treated sample; (**d**) S15 treated sample (same codes as in Table 2).

SEM images highlight the grafting and deposition of D-CD on cotton (Figure 5). Grafting was confirmed by the presence of D-CDs on samples S5 and S14. The D-CDs, having considerable lengths, can form bridges between neighboring cotton yarns (Figure 5b,c).

Deposition in certain parts of the fiber (Figure 5d) was probably followed by the penetration of D-CD inside the cotton; this fact, although difficult, was still possible and was confirmed by XRD analysis by changing the crystalline–amorphous ratio. The image of the S15 sample confirmed that D-CD can be deposited in the most accessible area of cotton, i.e., in the concave part of the bilobed shape (as is indicated by arrows in Figure 5d), from where, probably, a part of D-CD can enter inside the fiber; thus, they contribute to cotton yarn consolidation, conferring it multiple effects (wrinkle-proofing, hydrophilicity and antibacterial capacity).

The mechanism based on the formation of bridges or partial networks, both on the surface and inside the fiber, as a result of the phenomena of grafting and penetration of D-CD, was also confirmed by the XRD results.

#### 3.3.3. Energy Dispersive X-ray Spectroscopy (EDAX)

EDAX results from Table 3 confirmed the idea of D-CD presence on the treated samples, due to the increase of the percentage of O atoms and the appearance of N, Na and Cl atoms (Cl from MCT cycles that did not enter into the reaction). The atomic percentages of N (from MCT and ED) as well as of Cl that entered into the reaction were calculated using the following reasoning: in MCT-β-CD, there are 9 N atoms and 3 Cl and 3 Na atoms, therefore, the ratio is 3:1:1.

As N from the treated samples came from the 3 MCT cycles (9 atoms) and from ED (2 atoms), one can calculate the percent of N from MCT and ED using the atomic ratio. Cl percentage determined through EDAX analysis indicated how many percent of Cl atoms remained on the treated sample, i.e., how many did not react with cellulose. As it is known that Cl and Na are present in Cavasol W7 MCT with equal number of atoms, one can calculate the percent of Cl atoms that reacted by making the difference between the total number of chlorine atoms (equal to that of Na), minus the number of chlorine atoms not-involved in chemical reactions (that did not react).

#### 3.3.4. X-ray Diffraction Analysis (XRD)

**Figure 6:**
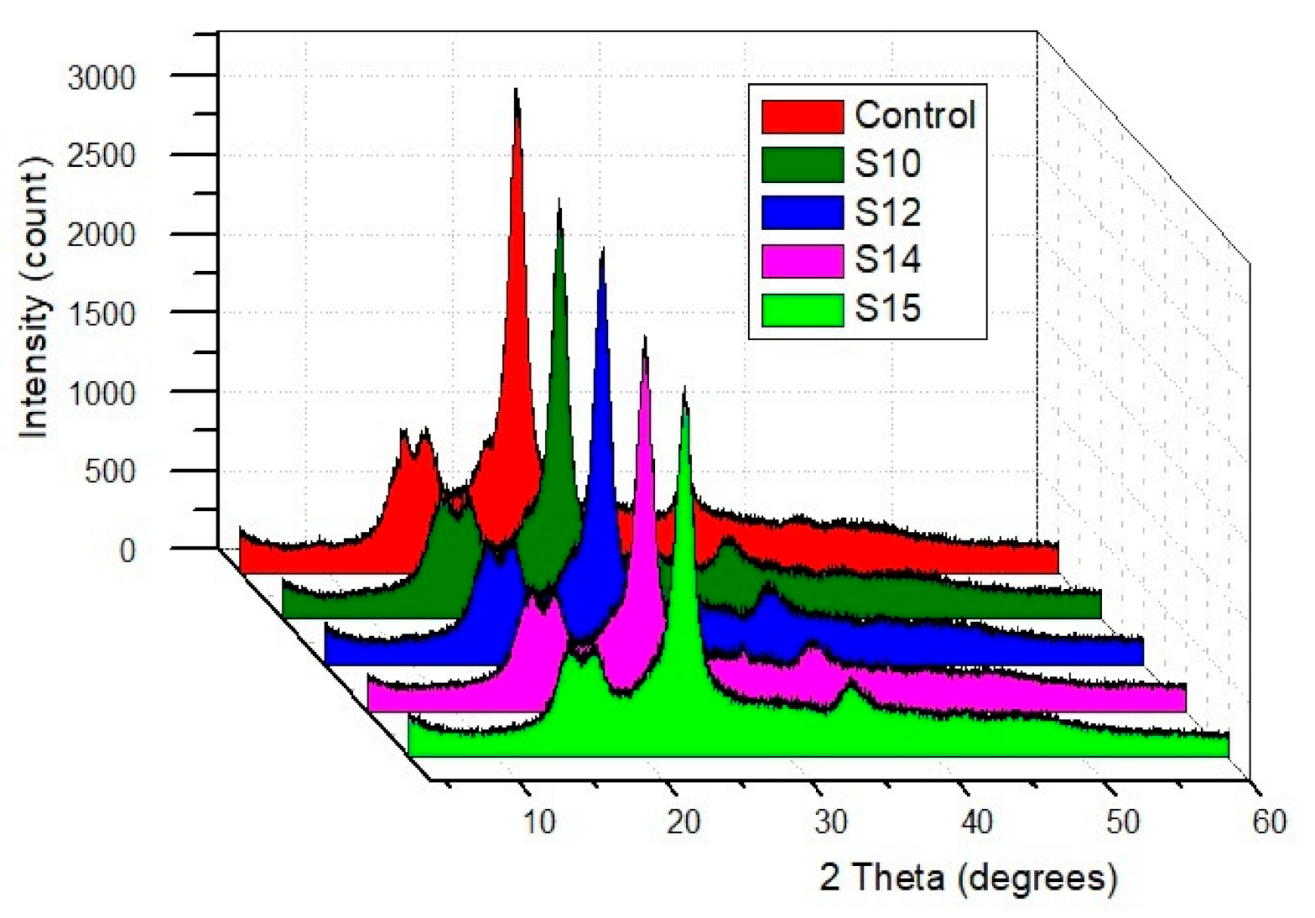
Differential scanning calorimetry (DSC) of control sample and some treated samples (S10, S12, S14 and S15).

The XRD spectra of the control sample (scoured cotton fabric) and four treated samples (S10, S12, S14 and S15) are shown in Figure 6.

The main peaks on each spectrum in Figure 6 appear around 14.9°, 16.6° and 22.9° for 2θ, and highlighted the presence of the crystalline zones in the cellulose Iβ. According to the literature, their corresponding Miller indices are (1–10), (110) and (200) [78,79]. Another moderate peak appeared near 34.3° for 2θ; this peak has Miller index (004) and is not a significant contributor of the value of crystallinity.

In the “Gaussian peak fitting with a linear background (Gaussian + linear)” method applied for computation of CrI index, the amorphous model was represented by an overlap of a linear fit and a wide Gaussian peak. This peak was stretched between 18° and 22° 2θ, being unobservable in Figure 6, because it was hidden under that rounded shoulder [44,79]. According to the literature, the linear fit is a part of the amorphous model because the scattering intensities are already corrected before the crystallinity analysis [44].

In general, a treatment that causes a greater degree of change in the internal order of the fiber leads to lower CrI values [80]. XRD results confirmed the penetration of D-CDs into cotton, determining the widening of amorphous zones, to the detriment of crystalline zones. This affirmation was proven by the smaller CrI values of the multifunctionalized samples in comparison to the control sample. The CrI indices for all samples included in Figure 6 had the following values: control sample (67.82%), S10 (65.10%), S12 (64.64%), S14 (62.46%) and S15 (60.18%).

### 3.4. Quantification Effects with MLR Analysis

MLR was used to quantify the following effects obtained by multifunctionalization of cotton: WRA_dry_, WRA_wet_ and water absorption capacity. Each mathematical model indicated very good adequacy (with Fisher’s test) and significant influences of X_1_, X_2_ and X_3_ on the studied effects (Student’s *t*-test).

#### 3.4.1. WRA for Dry and Wet Samples

The presence of the voluminous cycle of β-CD from the cross-bridges between the cellulose macromolecules ensures the necessary degree of stiffness and inflexibility of the long bridges between cellulose chains; this fact leads to weaker values concerning the anti-creasing effect and wrinkle recovering capacity, comparing to the effects produced by classical substances (of urea-formaldehyde type) used in wrinkle-proofing finishing [81]. This behavior induces the idea of D-CD penetration even inside cellulose fiber, being well known that WRA is smaller only when the treatment product adheres not to the external fiber layers, but to its core. So, each D-CD determines higher WRA_dry_ values comparative with the untreated sample (142 degrees). By applying the MLR method, in the case of WRA_dry_, Equation (3) was obtained:

(3) $${\mathrm{WRA}}_{\mathrm{dry}\;}=162.259+0.292X_{1}+1.33X_{2}+0.757X_{3}-3.306X_{1}X_{2}-1.631X_{1}X_{3\;}-\;0.368X_{2}X_{3}+14.500X_{1}^{2}+9.631{\;X}_{2}^{2}+7.386X_{3}^{2}$$

**Figure 7:**
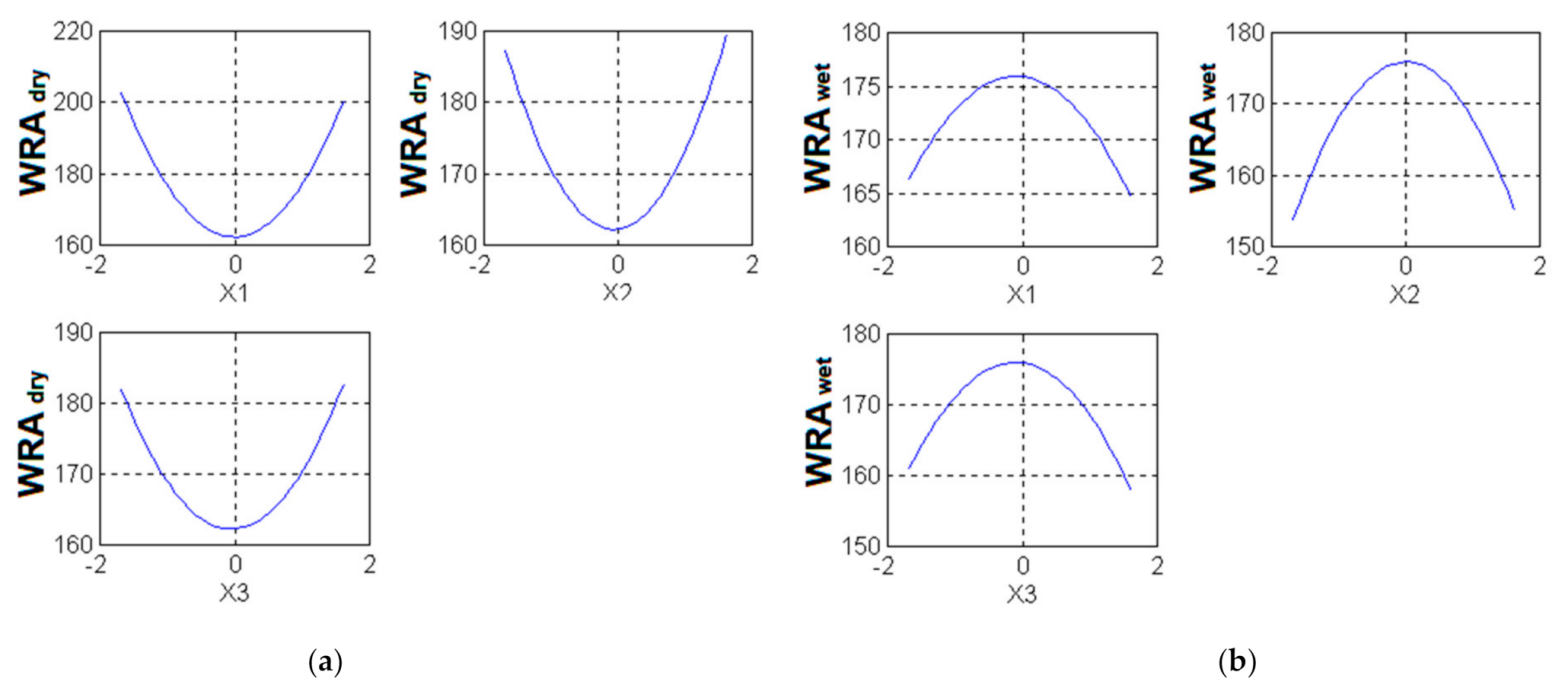
The variations of wrinkle recovery angle (WRA) function of the independent variables X_1_, X_2_ and X_3_.: (**a**) WRA_dry_; (**b**) WRA_wet_.

The sign (+) of the second order terms (Equation (3)), as well as Figure 7a also indicate a minimum for WRA_dry_ around the value coded with zero (X_1_ = −0.0216, X_2_ = −0.074 and X_3_ = −0.0555). These values correspond to 8.92% owf glyoxal, 8.73% owf ED and 7.45% owf MCT-β-CD. The increase of any independent variables (X_1_, X_2_, X_3_) above values coded with zero leads to the increase of WRA_dry_ values (Figure 7a).

By studying the level curves corresponding to the forms similar to the ellipsoids from Figure S3, it follows that the highest values for WRA_dry_ were obtained at 228 degrees.

All treated samples had higher WRA_wet_ values than the untreated sample (137 degrees). Equation (4) indicates the dependence WRA_wet_ = f (X_1_, X_2_, X_3_) as follows:

(4) $${\mathrm{WRA}}_{\mathrm{wet}\;}=175.888-0.773X_{1}-0.077X_{2}-1.307X_{3}+0.3X_{1}X_{2}+1.3X_{1}X_{3\;}+6.95X_{2}X_{3}-3.837X_{1}^{2}-7.884{\;X}_{2}^{2}-6.064X_{3}^{2}$$

The maximum values for WRA_wet_ (around 175 degrees in Figure 7b, easier seen in Figure S4) were obtained just in the center of the tested area, around the value coded with 0 for each of the three independent variables, i.e., X_1_ = −0.1326, X_2_ = −0.082 and X_3_ = −0.1692); the real values corresponded to 8.52% owf for glyoxal, 8.7% owf for ED and 7.35% owf for MCT-β-CD.

#### 3.4.2. Water Absorption Capacity

**Figure 8:**
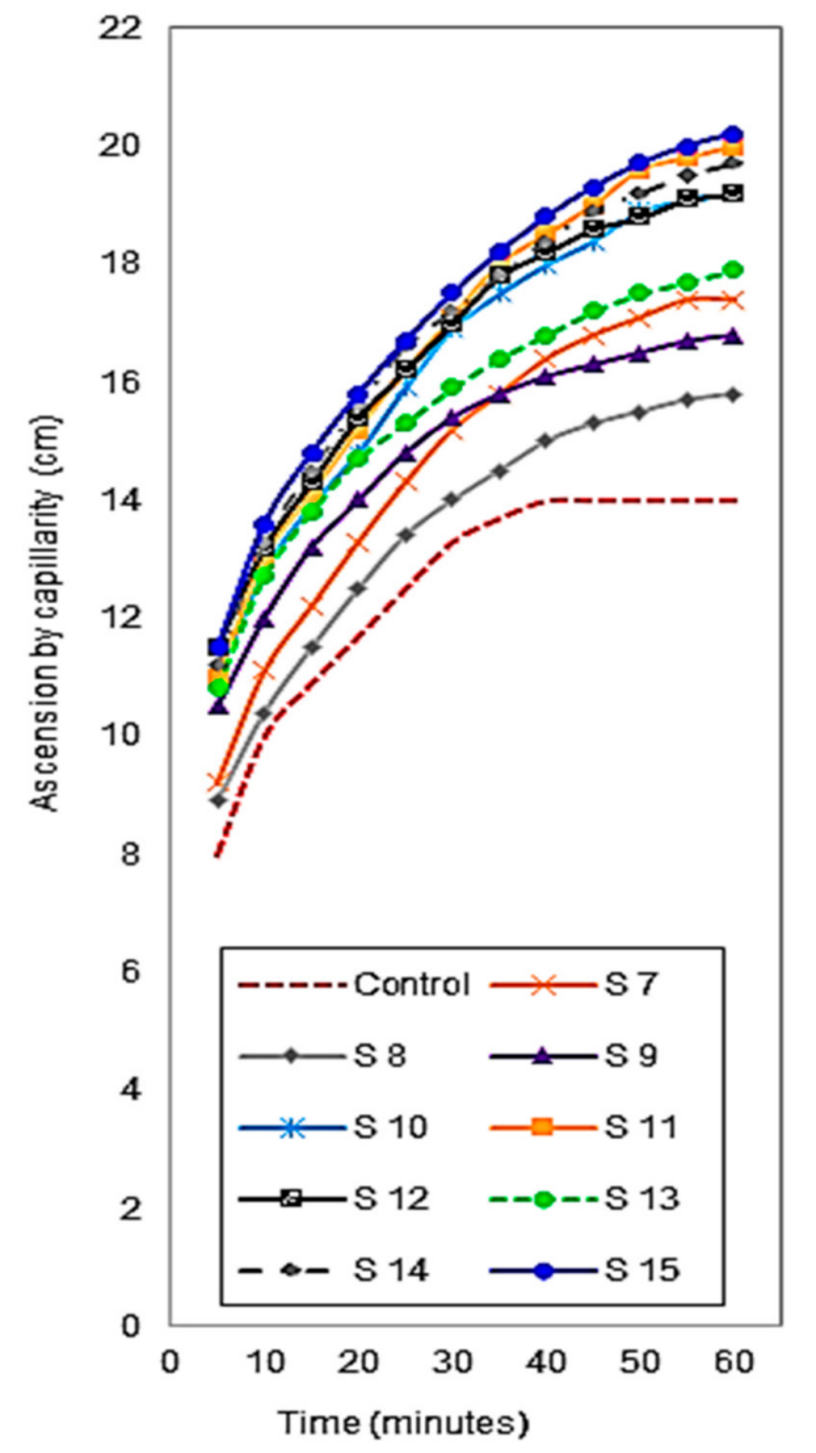
Curve of capillary ascension for some samples: control sample, S7–S15; (same codes as in Table 2).

**Figure 9:**
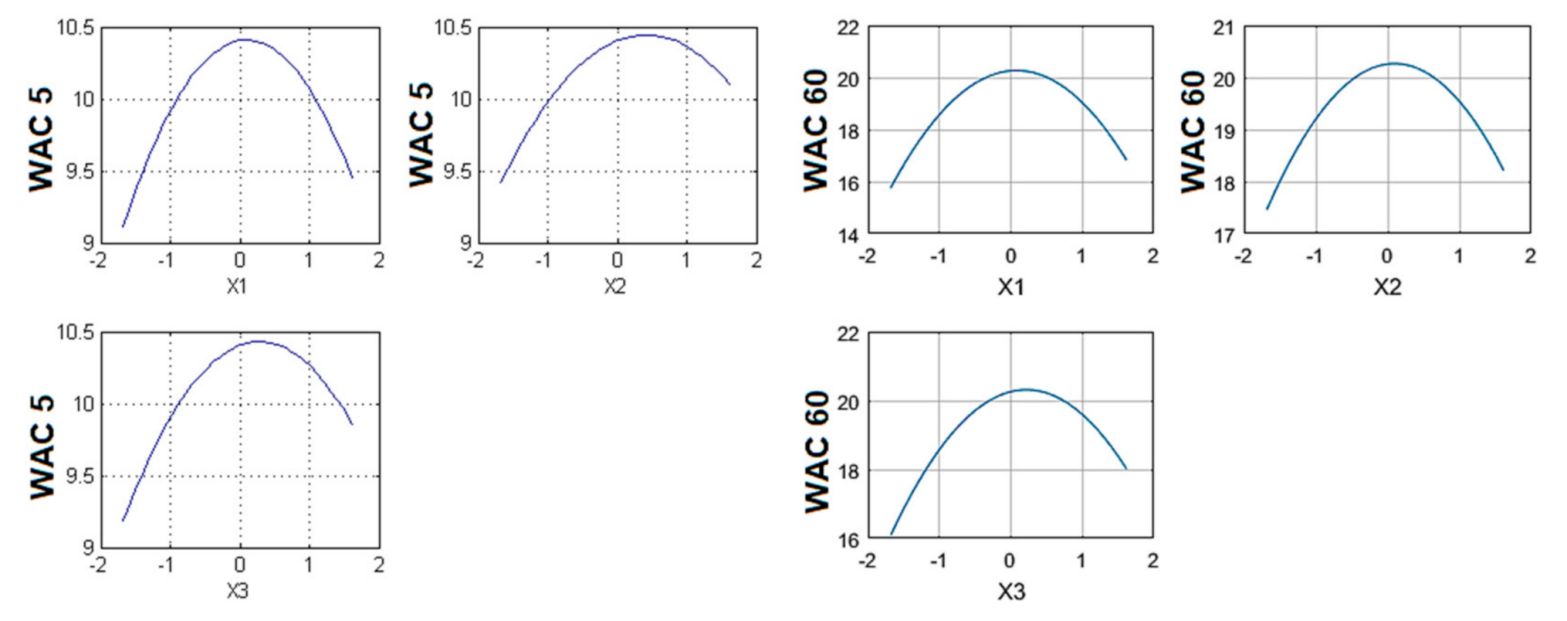
Water absorption capacity (WAC) (**a**) after 5 min; (**b**) after 60 min.

**Figure 10:**
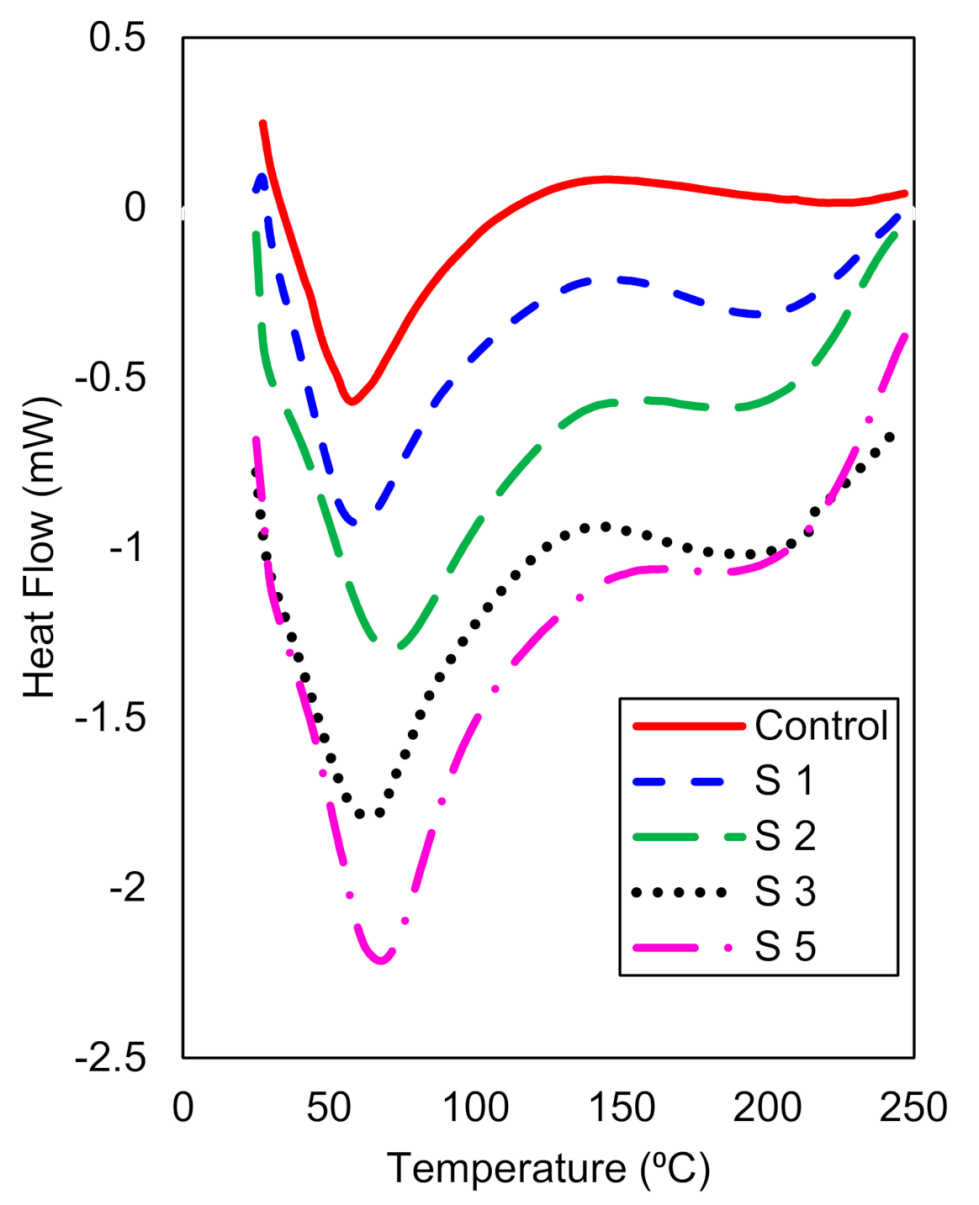
Differential scanning calorimetry (DSC) curves for control sample and some treated samples (S1, S2, S3 and S5; same codes as in Table 2).

In this paper, we used two methods to indicate the water affinity for the treated samples: water absorption by capillarity (Figure 8 and Figure 9) and DSC analysis (Figure 10).

Water affinity for treated cotton is the result of two phenomena: (1) rapid watering due to multiple H bonds formed between water molecules and the numerous polar groups of the D-CD; (2) the high capillarity of the treated cotton. The capillarity of cotton increased after treatment due to the partial/total entry into cotton of the D-CD that, having a large and irregular volume, led to the creation of cavities.

The phenomenon of capillary ascension was followed within an established time interval, and sample characterization was carried out through capillary ascension curve. For each tested sample, the cumulated increase of the liquid column height during one hour (every 5 min) was read. The results indicated that capillary absorption was realized at different rates within the studied interval, and that there was a stationary tendency toward the end of the 60 testing minutes (Figure 8).

After 60 testing minutes, the heights of the columns of solutions absorbed through capillarity varied between 14 and 20.2 cm. Comparing with control sample, the treated samples (samples S7 to S15) had a larger absorption capillarity, which confirmed the idea that by treating the cellulose with the solution of a voluminous product (D-CD, which contains several polar groups, OH type), one can reach significant absorption through capillarity. The coefficients of mathematical equations obtained with MLR are indicated in Table 4.

The stationary points (maximum points) (Figure 9 and Figure S5) are observed around values codified with zero (Table 5).

#### 3.4.3. Differential Scanning Calorimetry (DSC)

DSC results confirmed that treated samples had higher water absorption capacity. All the DSC curves (Figure 10) had an endothermic peak within the range 25–150 °C.

The size of the endothermic peak (indicated by dH values obtained by normalized integration) increases due to the increase of the energy necessary to evaporate the water existing in the treated samples or the water physically linked with cotton samples through hydrogen bonds [45,82]. The increase of the number of hydrogen bonds was confirmed by the shift of the temperature T_peak_ to higher values for all the treated samples, as compared to the control sample. The S1, S2, S3 and S5 samples had the following values for T_peak_: 59.5, 70.3, 61.1 and 67.3 °C. T_peak_ for the control sample was recorded at 57.3 °C. Sample S5 (compared with control sample and S1, S2, S3) had the higher value for the dehydration heat, dH = −132.03 J/g, which can be explained based on a higher MCT-β-CD concentration, which resulted in a larger number of hydrogen bonds between cotton and OH groups of β- cyclodextrin. The DSC curves for control sample and treated samples (S1, S2 and S3) indicate the following values for dH: −83.53, −93.46, −96.09 and −112.9 J/g.

This analysis indicated the presence of H-links or polar groups on treated/multifunctionalized samples; the increase in OH and NH_2_ groups indirectly indicated an increase in affinity for water or hydrophilicity of multifunctional cotton.

### 3.5. Influence of Treatment Conditions on the Textile Material Integrity

The treatment of cotton fabric with quite high concentrations of substances at temperatures of 150 °C imposed breaking strength testing.

It is known that for a good quality of cotton fabric after wrinkle-proofing treatment, one can accept a breaking strength loss of only one third from the initial value of the breaking strength of the control sample. This loss is due to the realization of the cross-linking phenomenon. The treatment did not result in decreases exceeding 30% for breaking strength, as follows from Table S3. In addition, some samples had breaking strengths greater than untreated cotton, which confirmed the dual mechanism based on the grafting and partial crosslinking.

Quality indices were calculated on both warp and weft directions. Table 6 presents the values of I_C_ index for treated samples according to the 15 treatment formulas. It can be noticed that I_C_ > 1 in all the cases, therefore, the treatment was considered very good, the loss of breaking strength being very small as reported to the obtained value of WRA.

### 3.6. Antibacterial Capacity

Antibacterial capacity is better when the diameter of the inhibition zone is higher; usually, the inhibition zones indicate the death of the microorganisms tested due to the destruction of their membranes [83].

**Figure 11:**
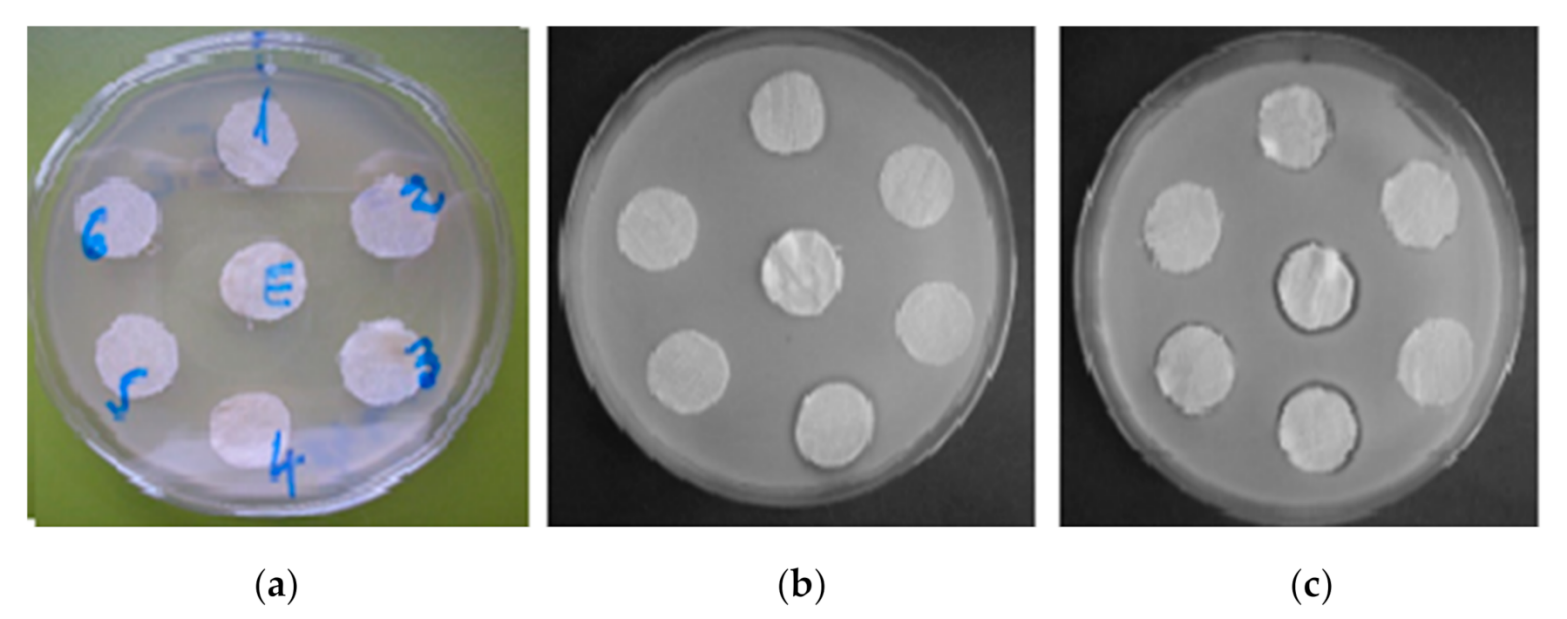
Images in natural light highlighting the antibacterial effects of samples S4 and S8 against *Micrococcus luteus*: (**a**) numbering on Petri dish lid: control sample (E), Sample S4 (**1**), Sample S4 washed 5 times (**2**), Sample S4 washed 10 times (**3**), Sample S8 (**4**), Sample S8 washed 5 times (**5**), Sample S8 washed 10 times (**6**); (**b**) after functionalization; (**c**) after functionalization and AgNO_3_ treatment on all 7 samples.

In Figure 11, the cotton samples had antibacterial capacities due to the multifunctionalization with D-CDs, in absence/presence of AgNO_3_. The antibacterial effects of the treated samples (S4, S8, S11 and S12) against *Escherichia coli* and *Micrococcus luteus* are shown in Supplementary Materials (Tables S4 and S5) and Table 7.

The diameter of the inhibition zone (Table 7) depends on the following aspects:

1. The type and structure of the microorganisms used for testing (different composition and ultrastructure of the cell wall of the two species).
2. The component of the multifunctional agent (D-CD) reflected by the proportions of the three agents (glyoxal, ED and MCT-β-CD) used in its synthesis. This aspect determined the differentiation between the antibacterial behavior against *Micrococcus luteus* of the samples functionalized with D-CDs: (a) The specimens/samples S4 and S8 that differed only by the content of MCT-β-CD (higher in sample S8) led to diameters of inhibition zones of 16 mm on S4 and 19 mm on S8). (b) The S12 sample was functionalized with a richer D-CD compound in ED and strongly inhibited the growth of *Micrococcus luteus* colonies (19 mm for the diameter of inhibition zone) when compared to the S11 sample (16 mm).
3. Applying or not applying additional treatment for antibacterial protection (with AgNO_3_).
4. Number of washing cycles after functionalization, respectively, after the functionalization treatment + AgNO_3_.

In the case of activity against *Escherichia coli*, all multifunctionalized samples were inactive, showing no inhibition zones; in exchange, for *Micrococcus luteus*, the diameter of inhibition zones on S4, S8, S11 and S12 samples varied between 16 and 19 mm. These treated cotton samples inhibited the growth of colonies of *Micrococcus luteus* because D-CDs have many chlorine atoms and free NH_2_ groups (especially in S8 and S12 samples).

The antimicrobial effects of the samples functionalized with D-CD did not persist after repeated washings (5–10 times), probably due to the formation of a complex between the functionalizing agent and the anion of the detergent used in the washing process [84].

Additional treatment with AgNO_3_ conducted on samples functionalized with D-CD modified both the inhibition zone size and durability to repeated washings.

The inhibition zones were even greater when the cotton samples functionalized with MCT-β-CD or with a derivative of β-CD were treated with AgNO_3_ [85]. The literature reported that a product enriched in NH_2_ and hydroxyl groups acquires very high affinity towards silver ions from AgNO_3_ [86].

A complex of Ag^+^/MCT-β-CD is formed; due to their large size, silver ions cannot enter the cyclodextrin cavity, but they will be chemically adsorbed in the crown of hydroxyl groups, thus remaining available at the entrance to the cyclodextrin cavity [83,85]. The silver ions can be detached from this architectonic structure and can act as a biocidal substance. Silver is capable of reacting with the proteins of the bacterial cell membrane and affect bacterial growth. In fact, silver ions can interact with phosphate-phosphorus nucleotides in DNA, resulting in interruption of its replication and inhibition of bacterial multiplication. They inhibit the activity of the enzyme/protein and damage the cell wall, which ultimately kills the microorganisms [85]. The diameters of the inhibition zones varied between 16 and 18 mm on the samples tested in *Escherichia coli* and between 16 and 20 mm in the case of *Micrococcus luteus*. The activity of S4 and S8 samples against *Micrococcus luteus* was strongly antibacterial, even after repeated washing, the bacillus was completely destroyed in the inhibition zone.

However, during washing, a certain amount of silver on the cotton samples is released into the wash water [83,87,88]. This transfer is carried out at different rates, thus, after 5 or 10 washing cycles, the amount of silver remaining on the cotton decreases more or less [89]. In the case of *Micrococcus luteus*, maintaining a high value of the inhibition zone even after repeated washings of multifunctionalized samples proved good fixation of silver and D-CD on cellulose fiber.

Some treated cotton samples had a diffuse aspect of the inhibition zone; this can be interpreted as a retardation of coccus development without complete elimination.

Samples functionalized with D-CD and treated with AgNO_3_ had antibacterial capacity against both *Escherichia coli* and *Micrococcus luteus*.

## 4. Conclusions

The novelty of this study consists in obtaining four simultaneous effects (wrinkle-proofing, hydrophilicity, antibacterial capacity and good integrity of the textile support) on a cotton fabric functionalized with a single compound, even if the first two effects are contradictory/antagonistic and were difficult to associate. It is known that the classic treatments of wrinkle-proofing usually lead to protection against wrinkles, strong stiffness of cotton, but also pronounced hydrophobia. This article demonstrates that this rule is canceled if the cotton fabric is treated with a suitable polyfunctional compound (D-CD), synthesized from three agents with high reactivity (glyoxal, ED and MCT-β-CD). The formation of D-CDs and its presence on cotton were rendered evident by several spectroscopic (^1^H-NMR, FTIR, EDAX, XRD) and thermoanalytic (DSC) analyses. Statistical analysis (MLR) of the results allowed to establish the mathematical equations according to which the effects obtained by multifunctionalization varied. The correlation between the optimal values of the obtained effects and the concentrations of the three agents used in D-CD synthesis was determined. It was found that D-CD containing 8.92% owf glyoxal, 8.73% owf ED and 7.45% owf MCT-β-CD led to the best wrinkle-proofing effects on dry cotton samples, WRA_dry_ being 228 degrees (control sample reaching only 142 degrees). The affinity of water for treated cotton was optimal in the case of the S15 sample which was functionalized with D-CD synthesized from 9.58% owf for glyoxal, 8.82% owf for ED and 6.53% owf for MCT-β-CD. D-CD functionalization of cotton samples gave them antibacterial effect against *Microccocus luteus*, recording 16–19 mm for the diameter of the inhibition zone; the application of a AgNO_3_ treatment improved antibacterial capacity against both *Escherichia coli* and *Microccocus luteus* (16–20 mm for the diameter of the inhibition zone) and improved the durability at repeated washings, confirming the stability of the silver fixation on the functionalized cotton samples. All these effects created by finishing treatments allowed the multifunctional cotton articles to ensure good comfort and protection of the human factor and can be used in various fields: from clothing and bed sheets to protective equipment in different sectors, including hospitals. The advantages of D-CD treatments are that they ennoble the cotton (they have quality indices I_C_ > 1) and, implicitly, the clothing products made from it, creating the possibility of wearing it immediately after washing and drying without prior smoothing.

## Figures and Tables

**Table 1:** Experiments coding.

Coding for X_1,_ X_2,_ X_3_	Independent Variables (Real Values)		
	X_1_ (% Owf)	X_2_ (% Owf)	X_3_ (% Owf)
−1.682	3	3	6
−1	5.4	5.4	6.6
0	9	9	7.5
+1	12.57	12.57	8.4
+1.682	15	15	9

**Table 2:** Experimental protocol.

X_1_ Code	X_2_ Code	X_3_ Code	D-CD Code	Code of Treated Sample
−1	−1	−1	D1	S1
1	−1	−1	D2	S2
−1	1	−1	D3	S3
1	1	−1	D4	S4
−1	−1	1	D5	S5
1	−1	1	D6	S6
−1	1	1	D7	S7
1	1	1	D8	S8
−1.682	0	0	D9	S9
+1.682	0	0	D10	S10
0	−1.682	0	D11	S11
0	+1.682	0	D12	S12
0	0	−1.682	D13	S13
0	0	+1.682	D14	S14
0	0	0	D15	S15
0	0	0	D16	S16
0	0	0	D17	S17
0	0	0	D18	S18
0	0	0	D19	S19
0	0	0	D20	S20

**Table 3:** EDAX results.

Sample Code	Element (% At) (By EDAX)					Element (% At) (By Computation)		
	C	O	N	Na	Cl ^a^	N ^b^	N ^c^	Cl ^d^
Control	54.62	45.38	-	-	-	-	-	-
S5	39.97	48.38	9.46	1.66	0.53	7.74	1.72	1.13
S14	34.05	54.04	9.79	1.89	0.22	8.01	1.78	1.67
S15	34.10	55.17	9.59	0.98	0.17	7.85	1.74	0.81

^a^ Chlorine not involved in chemical reactions; ^b^ N that comes from the triazinic cycle of MCT-β-CD. ^c^ N that comes from ED; ^d^ Chlorine involved in chemical reactions.

**Table 4:** The coefficients of mathematical equations obtained with multiple linear regression (MLR) for water absorption capacity after 5 to 60 min.

b_i_ Coefficient (i = 1 ÷ 3)	Water Absorption Capacity		
	5 min	30 min	60 min
b_o_	10.4044	16.2626	20.2586
b_1_	0.0762	0.0822	0.2297
b_2_	0.0192	0.2793	0.1710
b_3_	0.1819	0.5274	0.5086
b_12_	−0.0750	−0.0250	−0.1875
b_13_	−0.0750	−0.1500	−0.2125
b_23_	0.2250	−0.2750	0.2125
b_11_	−0.4104	−0.7210	−1.4540
b_22_	−0.2337	−0.4028	−0.8884
b_33_	−0.3221	−0.5796	−1.1712

**Table 5:** Stationary points for water absorption capacity.

WAC ^a^	Stationary Points	
	Codified Values	Real Values (% owf)
WAC 5	X_1_ = 0.1203	X_1_ = 9.4291
	X_2_ = 0.1675	X_2_ = 9.5875
	X_3_ = 0.4678	X_3_ = 7.9171
WAC 30	X_1_ = 0.1533	X_1_ = 9.5468
	X_2_ = 0.0247	X_2_ = 9.0881
	X_3_ = −0.9294	X_3_ = 6.6711
WAC 60	X_1_ = 0.16191	X_1_ = 9.5777
	X_2_ = −0.0512	X_2_ = 8.8173
	X_3_ = −1.0901	X_3_ = 6.5279

^a^ Water absorption capacity after 5–60 min.

**Table 6:** The values of index I_C_.

Sample Code	Treatment Conditions (Real Values)			I_C_ Quality Index	
	X_1_ (% Owf)	X_2_ (% Owf)	X_3_ (% Owf)	(On Warp)	(On Weft)
S1	5.4	5.4	6.6	1.14	1.01
S2	12.57	5.4	6.6	1.41	1.42
S3	5.4	12.57	6.6	1.37	1.46
S4	12.57	12.57	6.6	1.19	1.41
S5	5.4	5.4	8.4	1.25	1.16
S6	12.57	5.4	8.4	1.09	1.23
S7	5.4	12.57	8.4	1.07	1.35
S8	12.57	12.57	8.4	1.07	1.39
S9	3	9	7.5	1.01	1.49
S10	15	9	7.5	1.06	1.17
S11	9	3	7.5	1.26	1.01
S12	9	15	7.5	1.03	1.04
S13	9	9	6	1.00	1.00
S14	9	9	9	1.03	1.07
S15	9	9	7.5	1.04	1.01

**Table 7:** Antibacterial activity against *Micrococcus luteus* and *Escherichia coli*.

Sample	Diameter of Inhibition Zone (mm)			
	*Escherichia coli* *DSMZ 498*		*Micrococcus luteus* *ATCC 934*	
	*Without* *AgNO_3_*	*With* *AgNO_3_*	*Without* *AgNO_3_*	*With* *AgNO_3_*
S4	0	17	17	18
S4 washed 5 times	0	0	0	17
S4 washed 10 times	0	0	0	16
S8	0	18	19	20
S8 washed 5 times	0	17	0	18
S8 washed 10 times	0	0	0	16
S11	0	17	16	-
S11 washed 5 times	0	16	0	-
S11 washed 10 times	0	0	0	-
S12	0	16	19	-
S12 washed 5 times	0	0	0	-

## Data Availability

All data involved in this study have been included in the text of this article and in the supplementary material.

## Supplementary Materials

The following are available online at https://www.mdpi.com/2073-4360/13/3/410/s1, Table S1: Chemical structures of the utilized substances; Figure S1: Structure of β-cyclodextrin: (a) dimensions of toroid structure; (b) chemical structure of β-CD; (c) β-CD arrangement which generates a toroid form; Table S2: The properties of β-cyclodextrin; Figure S2: Getting a multifunctional cotton, in detail; Figure S3: Graphical dependence of WRA _dry_ on independent variables (X_1_, X_2_ and X_3_) and level curves (their contour); Figure S4: Graphical dependence of WRA _wet_ on independent variables (X_1_, X_2_ and X_3_) and level curves (their contour); Figure S5: 3D graphical dependence of WAC on X_1_, X_2_ and X_3_ and level curves (their contour); Table S3: Breaking strength along the warp and weft directions; Table S4: Showing off the antibacterial effects of the samples S4 and S8 against *Escherichia coli* and *Micrococcus luteus*; Table S5: Showing the antibacterial effects of the samples S12 and S11 against *Escherichia coli* and *Micrococcus luteus.*
