# Supplementary material for: Multifunctional Finishing of Cotton with Compounds Derived from MCT-β-CD and Quantification of Effects Using MLR Statistical Analysis
Source: Polymers (Basel). 2021 Jan 27;13(3):410. doi: 10.3390/polym13030410 (PMC7865583; doi:10.3390/polym13030410)
Supplement: Supplementary file 1 [file polymers-13-00410-s001.pdf]

Supplementary Materials

for

Multifunctional finishing of cotton with compounds derived from MCT-β-CD and quantification of effects using MLR statistical analysis

Vasilica Popescu[1*], Marioara Petrea[2] and Andrei Popescu[3]

[1] Department of Chemical Engineering in Textiles and Leather, "Gheorghe Asachi" Technical University of Iasi, 700050, Romania; e-mail: vpopescu65@tex.tuiasi.ro
[2] Department of Wood Processing and Design of Wood Products, "Transilvania" University of Brasov, 500068, Romania; e-mail: gmaria@unitbv.ro
[3] Department of Machine Design, Mechatronics and Robotics, "Gheorghe Asachi" Technical University of Iasi, 700050, Romania; e-mail: andrei.popescu@academic.tuiasi.ro
* Correspondence: vpopescu65@tex.tuiasi.ro

*Correspondence: vpopescu65@tex.tuiasi.ro

Characterization of the reagents

a) **Chemical structures of reagents**

b) **Characterization of β-cyclodextrin: chemical structure and properties**

Chemical reaction of D-CD with cellulose

Results of statistical analysis

Influence of treatment conditions on textile material integrity

Antibacterial capacity

References

Characterization of the reagents

a) Chemical structures of reagents

Table S1. Chemical structures of the utilized substances

Substance name		Chemical	Average molecular weigh (g/mol)
**Non-hydrated form of glyoxal**	Glyoxal (monomer (M), a*nhydrous form*)	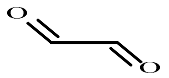	58
**The monohydrated (MH) and dihydrates (DH) forms of 40 % glyoxal solution:**	Monohydrated glyoxal (MH)	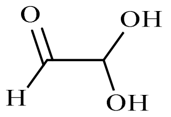	76
	Ethane-1,1,2,2-tetraol (or bis gem-diol) (DH)	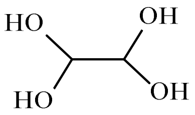	94
	Dimer type dioxalane: (1,3) dioxolane-4,5-trans-diol) (MH)	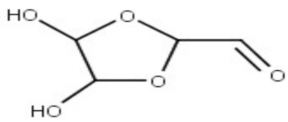	150
	Dimer type dioxalane (i.e.2-dihydroxymethyl-(1,3) dioxolane-4,5-trans-diol) (DH)	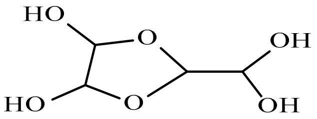	152
	Trimer: 2,2'-bi-1,3-dioxolanyl-4,4',5,5'tetraol (DH)	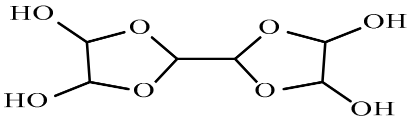	210
**Ethylenediamine**	ED	$H_2N - CH_2 - CH_2 - NH_2$	60
**Monochlorotriazinyl-β-cyclodextrin or Cavasol W7 MCT or Cavatex W7 MCT**	MCT-β-CD	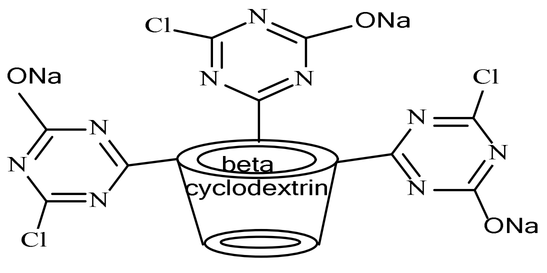	1560 for DS=2.8

b) Characterization of β-cyclodextrin: chemical structure and properties

The β-cyclodextrin (also named Schardinger dextrin, cycloglucose, cycloamylose, cycloglucoamylose) is a cyclic non-reductive oligosaccharide consisting of 7 α-D (+)- glucopyranosyl, α-1,4 glycosidic connected units. The 7 units are arranged as a truncated cone empty inside (Figure S1). The hydroxyl groups are directed as follows: the secondary groups which are directly attached to glucopyranosyl ring are rigid, while the primary groups, O(6)H can rotate around the C(5)- C(6) bonds,

adopting the (-) or (+) *gauche* directions. The compound β-cyclodextrin contains 21 hydroxyl groups, whose reactivity decreases in the sequence O (6) H > O (2) H > O (3) H. Hydroxyl groups can participate in various chemical reactions (etherification, esterification, sulphonation).

Detailed characterization of β-cyclodextrin is presented in Table S2. The main structural characteristics of β-cyclodextrin [1-5] are shown in Figure S1.

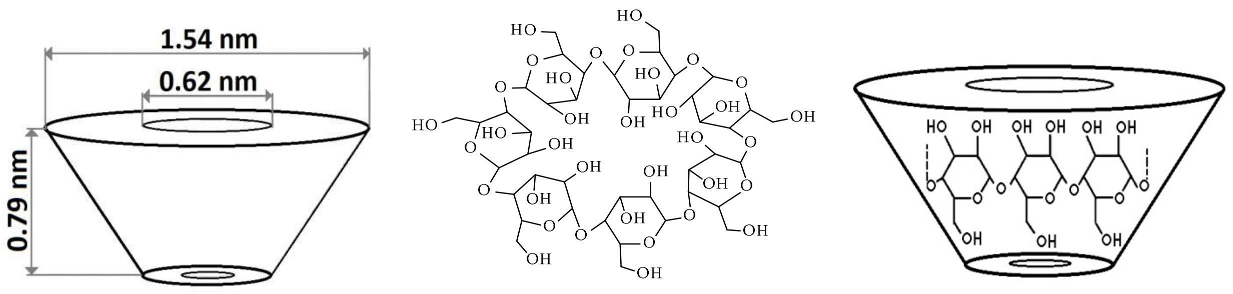

(a) **(b)** **(c)**

Figure S1. Structure of β-cyclodextrin: a) dimensions of toroid structure; b) chemical structure of β-CD; c) β-CD arrangement which generates a toroid form.

Table S2. The properties of β-cyclodextrin

**β-cyclodextrin properties**	**Values for the β-cyclodextrin properties**
Molecular mass	1135
Number of glucosidic units	7
Diameter of the inner cavity (nm)	0.62
Outer diameter of macrocycle	1.54
Conoid height (nm)	0.79
Water solubility (g/100mL.25°C)	18.5
Surface tension (mN/m)	71
Melting point (^{o}C)	255-265
Crystallization in water	13-15
Water molecules in cavities	11

Chemical reaction of D-CD with cellulose

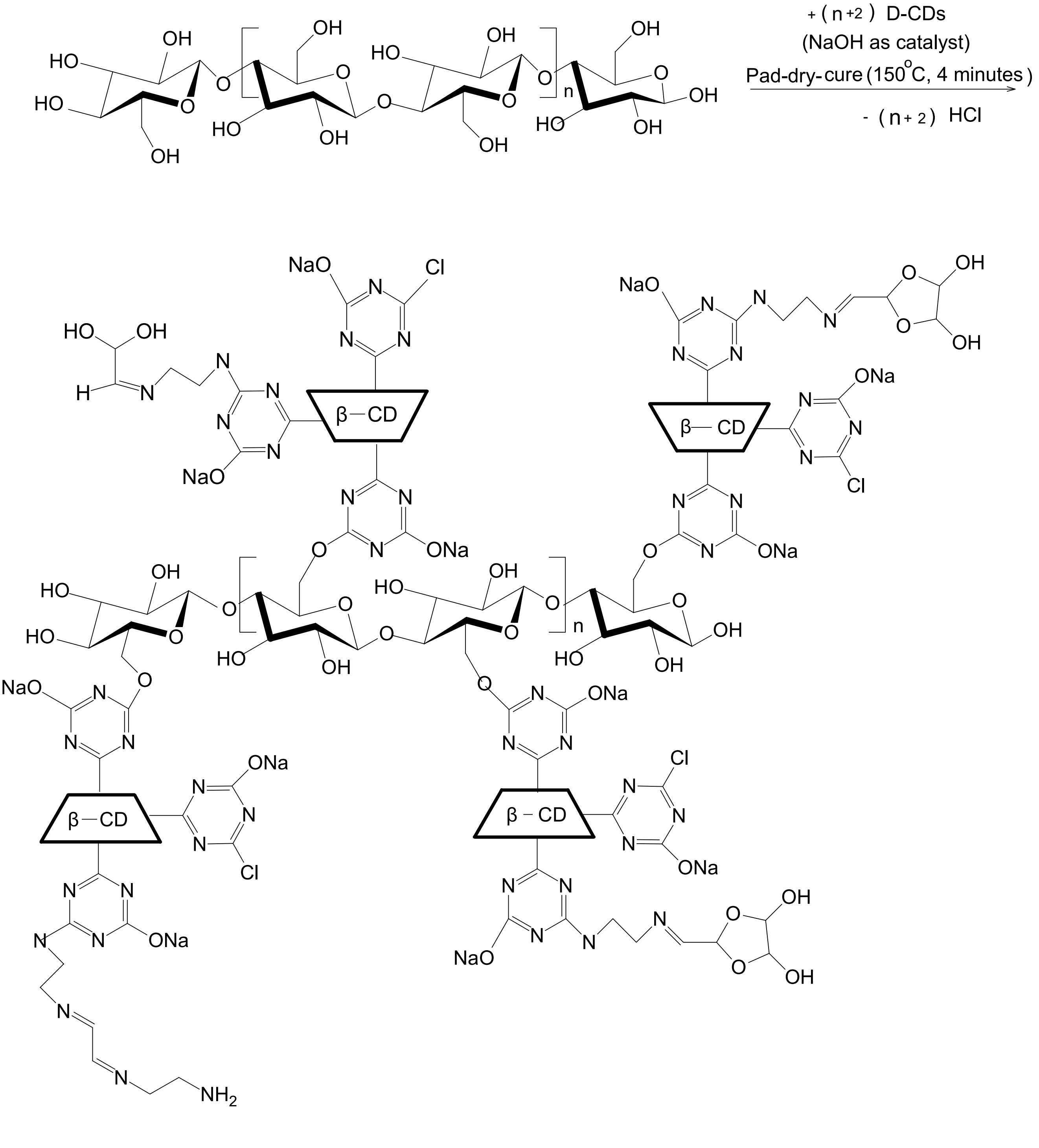

Figure S2. Getting of a multifunctional cotton, in detail.

Results of statistical analysis

WRA dry

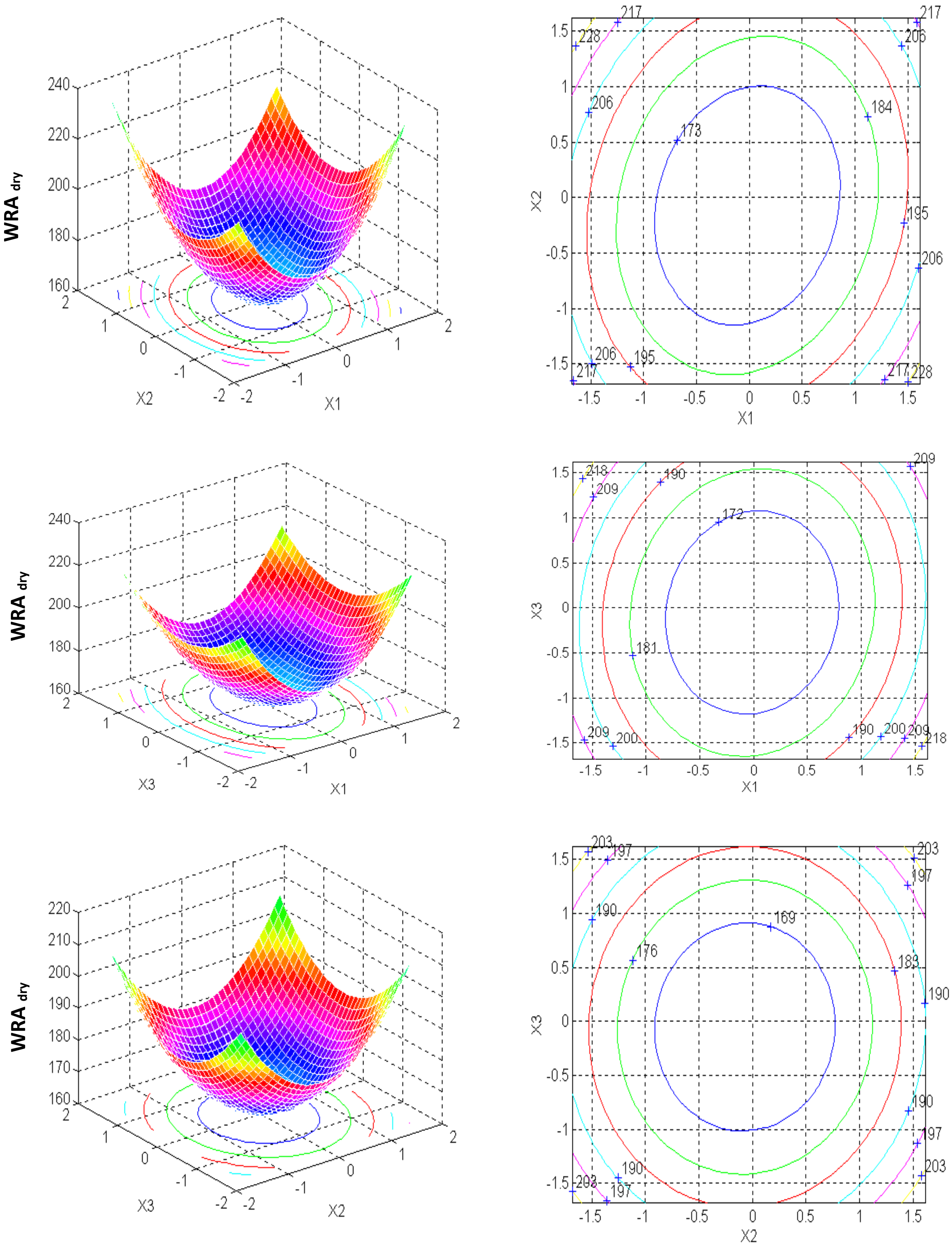

Figure S3. Graphical dependence of WRA dry on independent variables (X_1, X_2 and X_3) and level curves (their contour).

WRA wet

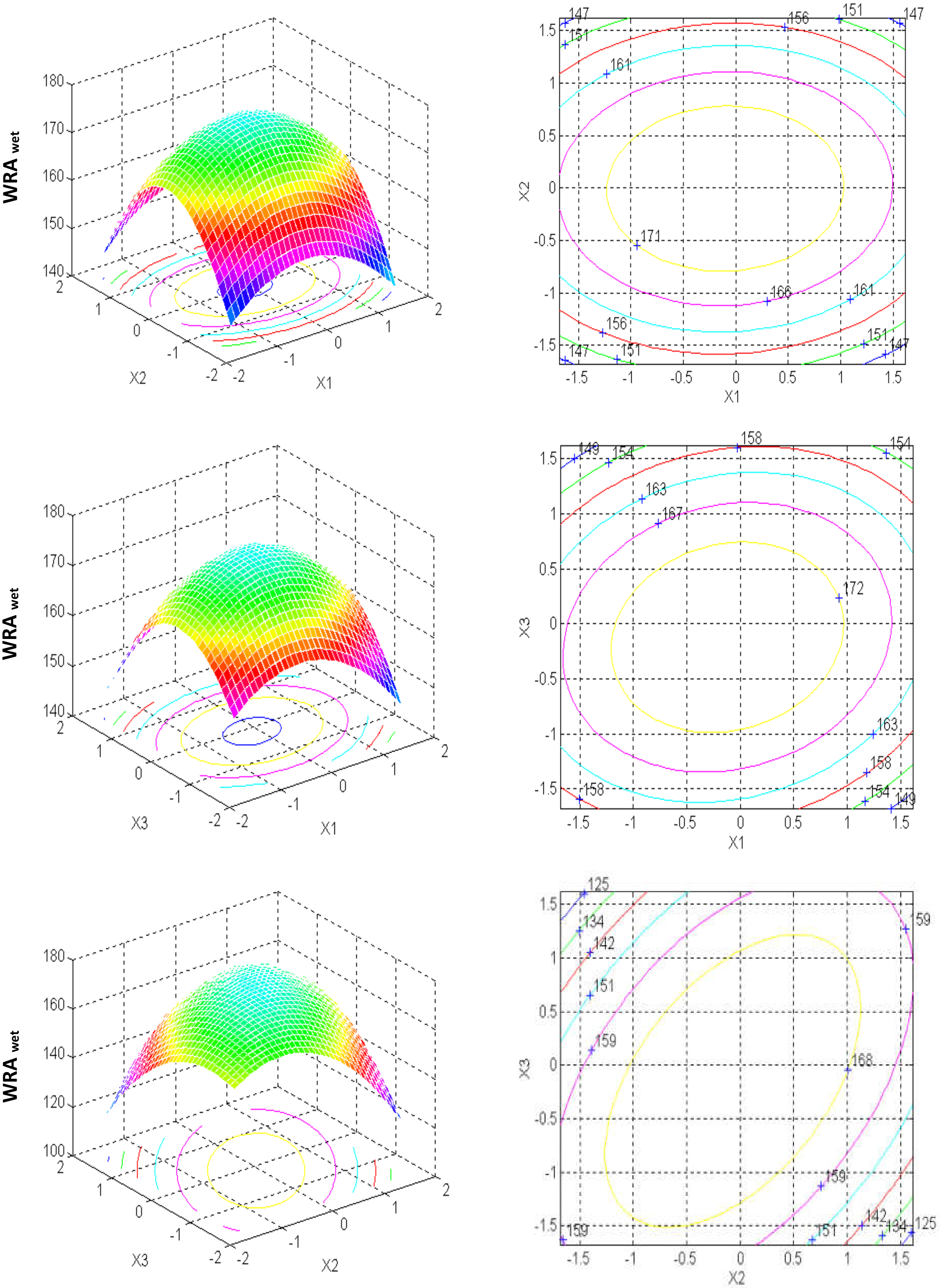

Figure S4. Graphical dependence of WRA wet on independent variables (X_1, X_2 and X_3) and level curves (their contour).

Water absorption capacity (WAC)

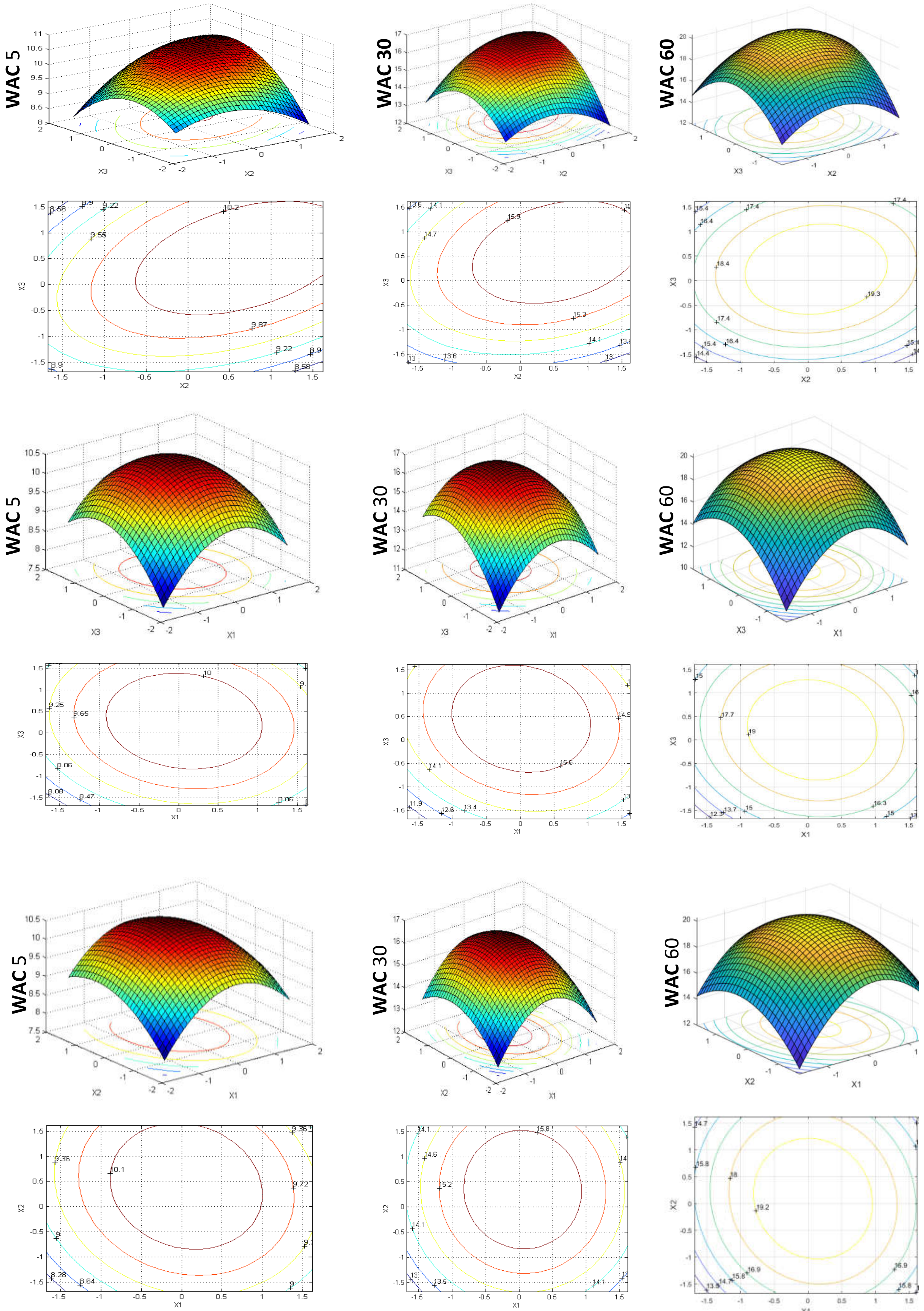

Figure S5. 3D graphical dependence of WAC on X_1, X_2 and X_3 and level curves (their contour).

Influence of treatment conditions on textile material integrity

Table S3. Breaking strength along the warp and weft directions

**Sample**	**Treatment conditions (Codes)** X1	X2	X3	**Treatment conditions (Real values)** X1 (% owf)	X2 (% owf)	X3 (% owf)	**Breaking strength (N)** *On warp*	*On weft*
Control	0	0	0	0	0	0	424.12	364.0
S1	-1	-1	-1	5.4	5.4	6.6	428.5	339.2
S2	1	-1	-1	12.57	5.4	6.6	471.5	449.5
S3	-1	1	-1	5.4	12.57	6.6	465.0	420.0
S4	1	1	-1	12.57	12.57	6.6	408.5	446.5
S5	-1	-1	1	5.4	5.4	8.4	435.0	380.8
S6	1	-1	1	12.57	5.4	8.4	398.5	406.5
S7	-1	1	1	5.4	12.57	8.4	392.8	460.5
S8	1	1	1	12.57	12.57	8.4	374.0	414.5
S9	-1.682	0	0	3	9	7.5	354.8	436.0
S10	+1.682	0	0	15	9	7.5	406.5	354.0
S11	0	-1.682	0	9	3	7.5	434.0	344.8
S12	0	+1.682	0	9	15	7.5	409.5	347.6
S13	0	0	-1.682	9	9	6	435.5	324.4
S14	0	0	+1.682	9	9	9	400.0	348.4
S15	0	0	0	9	9	7	438.0	349.6

Antibacterial activity

Table S4. Showing off the antibacterial effects of the samples S4 and S8 against *Escherichia coli and Micrococcus luteus*

**Treatment**	**Escherichia coli (DSMZ 498) (Gram-negative bacillus)**	**Micrococcus luteus (ATCC 934) (Gram-positive coccus)**
a) Without AgNO3 treatment<br><br>Sample codes:<br>E = Control sample<br>1′ = Sample S4<br>2′ = Sample S4 washed 5 times<br>3′ = Sample S4 washed 10 times<br>4′ = Sample S8<br>5 ′= Sample S8 washed 5 times<br>6′ = Sample S8 washed 10 times	Numbering on Petri dish lid<br>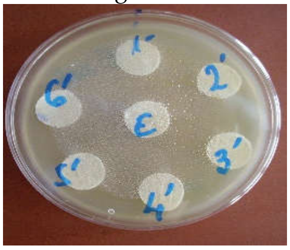<br>Image in strong artificial light<br>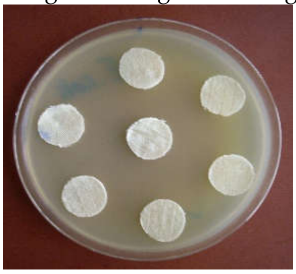	Numbering on Petri dish lid<br>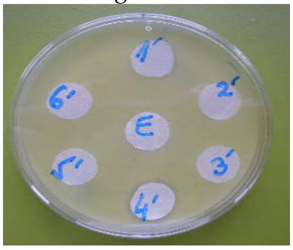<br>Image in strong artificial light<br>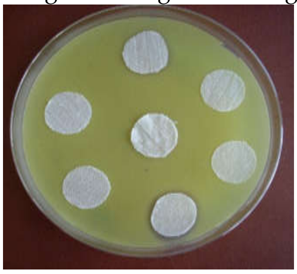<br>Image in natural light<br>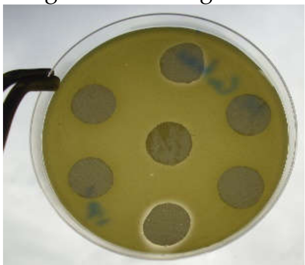
a) With 5g/L AgNO3 treatment<br><br>Sample codes:<br>E=Control sample (treated with AgNO3)<br>1 = Sample S4<br>2 = Sample S4 washed 5 times<br>3 = Sample S4 washed 10 times<br>4 = Sample S8<br>5 = Sample S8 washed 5 times<br>6 = Sample S8 washed 10 times	Numbering on Petri dish lid<br>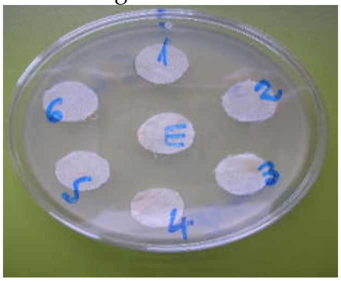<br>Image in strong artificial light<br>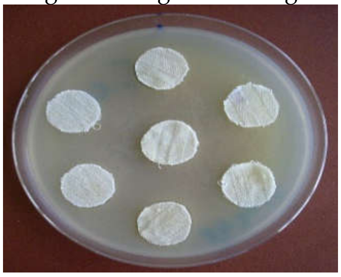<br>Image in natural light<br>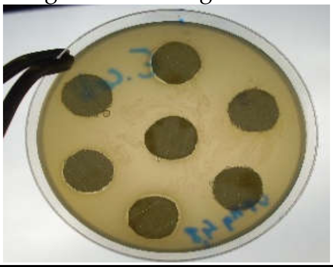	Numbering on Petri dish lid<br>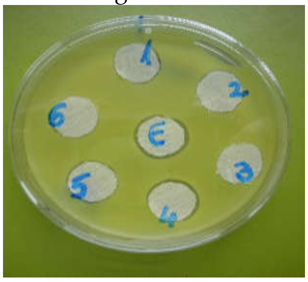<br>Image in strong artificial light<br>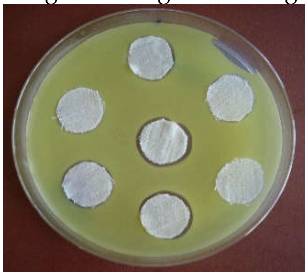<br>Image in natural light<br>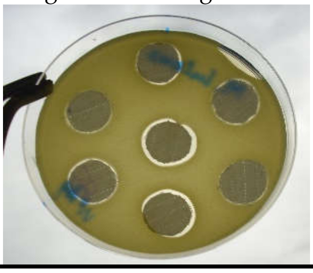

Table S5 Showing off the antibacterial effects of the samples S12 and S11 against *Escherichia coli and Micrococcus luteus*

**Treatment**	**Escherichia coli (DSMZ 498) (Gram-negative bacillus)**	**Micrococcus luteus (ATCC 934) (Gram-positive coccus)**
a) Without AgNO3 treatment<br><br>Sample codes:<br>E = Control sample<br>1 = Sample S11<br>2 = Sample S11 washed 5 times<br>3 = Sample S11 washed 10 times<br>4 = Sample S12<br>5 = Sample S12 washed 5 times<br>6 = Sample S12 washed 10 times	Numbering on Petri dish lid<br>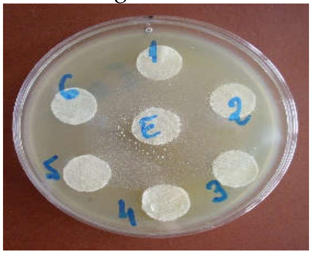<br>Image in strong artificial light<br>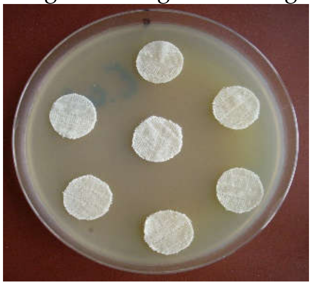	Numbering on Petri dish lid<br>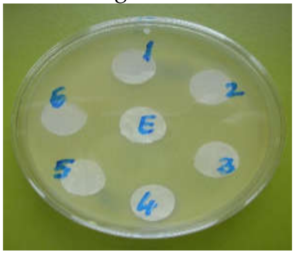<br>Image in strong artificial light<br>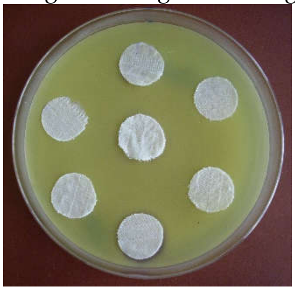<br>Image in natural light<br>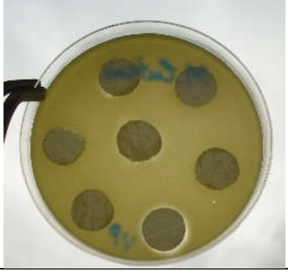
b) With 5g/L AgNO3 treatment<br><br>Sample codes:<br>E = Control sample (treated with $AgNO_3$)<br>1 = Sample S11<br>2 = Sample S11 washed 5 times<br>3 = Sample S11 washed 10 times<br>4 = Sample S12<br>5 = Sample S12 washed 5 times	Numbering on Petri dish lid<br>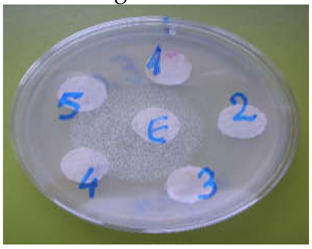<br>Image in strong artificial light<br>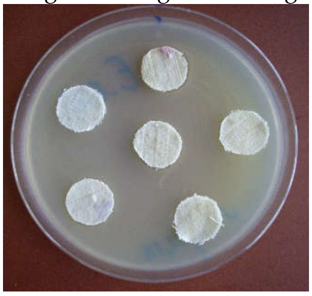<br>Image in natural light<br>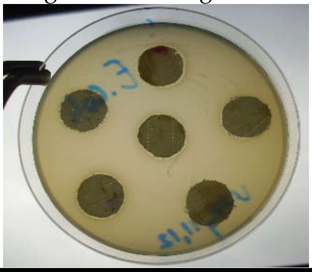	

References*

1. Wacker Chemie GmbH. Technical Information Sheet CAVATEX W7 MC, **2003.**
2. Szejtli, J. Cyclodextrins and their inclusion complexes. Budapest: Akademiai Kiado, **1982.**
3. Szejtli, J. Introduction and General Overview of Cyclodextrin Chemistry. *Chemical Reviews* **1998,** *98*, 1743-1754
4. Szejtli, J. Utilization of cyclodextrins in industrial products and processes. *J. Mater. Chem.*, **1997**, *7*, 575-587.
5. Saenger, W.; Steiner, T. Cyclodextrin Inclusion Complexes: Host-Guest Interactions and Hydrogen-Bonding Networks. *Acta Cryst.* **1998**, *A54*, 798-805.

**the references correspond to numbers 34-38 in the manuscript*
